# Automated sequence-level analysis of kinetics and thermodynamics for domain-level DNA strand-displacement systems

**DOI:** 10.1098/rsif.2018.0107

**Published:** 2018-12-19

**Authors:** Joseph Berleant, Christopher Berlind, Stefan Badelt, Frits Dannenberg, Joseph Schaeffer, Erik Winfree

**Affiliations:** California Institute of Technology, Pasadena, CA, USA

**Keywords:** DNA strand displacement, formal verification, nucleic acid secondary structure, kinetics, thermodynamics

## Abstract

As an engineering material, DNA is well suited for the construction of biochemical circuits and systems, because it is simple enough that its interactions can be rationally designed using Watson–Crick base pairing rules, yet the design space is remarkably rich. When designing DNA systems, this simplicity permits using functional sections of each strand, called domains, without considering particular nucleotide sequences. However, the actual sequences used may have interactions not predicted at the domain-level abstraction, and new rigorous analysis techniques are needed to determine the extent to which the chosen sequences conform to the system’s domain-level description. We have developed a computational method for verifying sequence-level systems by identifying discrepancies between the domain-level and sequence-level behaviour. This method takes a DNA system, as specified using the domain-level tool Peppercorn, and analyses data from the stochastic sequence-level simulator Multistrand and sequence-level thermodynamic analysis tool NUPACK to estimate important aspects of the system, such as reaction rate constants and secondary structure formation. These techniques, implemented as the Python package KinDA, will allow researchers to predict the kinetic and thermodynamic behaviour of domain-level systems after sequence assignment, as well as to detect violations of the intended behaviour.

## 1. Introduction

DNA is a widely used engineering substrate for biochemical circuits and systems. Using simple Watson–Crick base-pairing rules, molecules can be designed to fold into stable conformations and large assemblies [1], but they can also be programmed to implement dynamic systems using toehold-mediated DNA strand displacement [2] for triggered rearrangement of molecular components [3]. Experimental demonstrations have shown that DNA-based circuits can carry out a diverse range of information-processing tasks, including amplification and analogue computation [4–12], digital logic gates and circuits [13–18], neural network pattern recognition [19–21], probabilistic circuits [22] and the implementation of chemical reaction network (CRN) dynamics [23,24]. Theoretical studies have established that DNA-based circuits are capable of arbitrarily complex digital and analogue circuits [25–27], efficient neural network computation and autonomous learning [28,29], the full range of dynamical behaviours supported by mass-action kinetics of abstract CRNs [30–32], and even the full range of algorithmic behaviours supported by Turing machines [33,34].

DNA-based circuits can be large and complex, involving interactions between many DNA molecules each composed of multiple interacting DNA strands. Experimentally demonstrated systems have involved hundreds of synthesized molecules with thousands of potential interactions [16,19,21]. Design of these systems can be a time-consuming process because the sequence and length of every DNA strand must be carefully chosen to tune the rate of each reaction, as well as to avoid interactions between system components that should be orthogonal. This paper focuses on the non-trivial problem of system *verification*, that is, checking that a system as a whole behaves as designed. As DNA-based systems grow in size and complexity, there is an increasing need within the nucleic acid programming community for a unified framework to analyse and verify arbitrary DNA systems.

The design and verification of DNA systems is often initially performed without regard to specific DNA sequences by describing systems using *domains*, functionally distinct contiguous sections composing each DNA strand. Under certain idealized assumptions about interactions between domains, it is possible to verify the system by enumerating all possible reactions between domain-level DNA complexes [36–38] and establishing a correspondence with a formal description of the intended circuit function [39–42].

Domain-level analysis may be contrasted with sequence-level analysis, which must account for additional non-ideal interactions between domains, such as binding due to partial domain sequence matches. Several software packages are available for performing sequence-level analysis without reference to the system’s intended behaviour, both with respect to thermodynamic equilibrium [43–45] and with respect to kinetic pathways [46–48]. Such de novo analysis can uncover completely unexpected system behaviour, but this analysis can be intractable with more complex systems.

We present a novel framework for analysing and verifying an important subset of DNA systems: unpseudoknotted strand-displacement systems designed using domains. In contrast to previous sequence-level techniques, our framework aims to analyse entire systems rather than individual pathways or collections of small numbers of molecules, while still giving users access to detailed information about the behaviour of a system’s components to debug potential problems. This analysis is made feasible by using the domain-level system description to guide sequence-level analysis so that the behaviour of the sequence-level system can be verified by comparing against domain-level predictions.

Section 2 describes basic concepts and current methods of analysing a system at the domain and sequence levels. In §3, we propose a conceptual framework that augments existing sequence-level analysis techniques by using the domain-level information to guide stochastic simulations and thermodynamic analysis. Section 4 describes four case studies that demonstrate the use of this framework on representative DNA strand-displacement (DSD) schemes. The framework described in this paper has been implemented in the Python software package KinDA (Kinetic DNA strand-displacement analyser), available on GitHub [35] and via a pre-built Amazon Machine Image.

## 2. Background

### 2.1. Basic concepts

**Figure 1.**
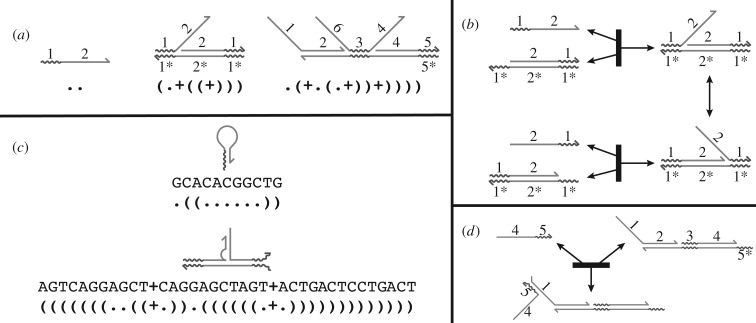
Overview of DNA systems at the domain and sequence level. (*a*) Examples of domain-level secondary structure, specified in domain-level dot-parens-plus form. Domain-level dot-parens-plus representations use a period ‘.’ to represent an unbound domain, a balanced pair of parentheses ‘(’ and ‘)’ to specify each pair of bound domains, and the beginning of a new strand with a plus ‘ + ’. (*b*) A simple domain-level reaction termed toehold-mediated strand-displacement, in which an invading strand (domains 1 and 2) binds to a base strand and displaces the incumbent strand (domains 2 and 1). The toehold (domain 1) is shorter than domain 2; two strands bound merely by a toehold may dissociate spontaneously. (*c*) In sequence-level dot-parens-plus notation, each character corresponds to a nucleotide rather than a domain. Owing to unintended binding between non-complementary domains, sequence-level conformations may be quite different from the designed domain-level conformation. Illustrated is hairpin formation in a strand that is intended to have no structure, and an intermediate of branch migration in which the tails have a spurious interaction and a helix end frays. (*d*) At the sequence level, additional interactions are possible due to partial binding between complementary and non-complementary domains. Illustrated is an unproductive reaction that involves fleeting spurious binding between domains that are not designed to be complementary.

A domain-level description of a DNA system represents the strands and complexes in terms of domains rather than specific nucleotide sequences. Each set of bound DNA strands, or *complex*, exhibits a particular secondary structure. A valid secondary structure must have each domain unbound, or completely bound to a single complementary domain. In this paper, we further dictate that valid secondary structures be non-pseudoknotted (i.e. have a well-defined dot-parens-plus representation [49]). Complementary domains are denoted throughout this paper with an asterisk (*). Examples of valid structures are shown in figure 1*a*, with accompanying dot-parens-plus structure representations [45].

DNA interactions that generate new complexes or changes in secondary structure are called *reactions*. Multiple sequential reactions can perform an essential molecular primitive called toehold-mediated strand displacement, in which two complexes bind at a short domain, or *toehold*, which makes a subsequent branch migration step favourable (figure 1*b*). Additional molecular primitives are also available for incorporation into DNA systems: hybridization of complementary single-stranded domains to form a duplex, unbinding of a duplex region for sufficiently short domains, and strand exchange by four-way branch migration at a branched junction. Systems built using any combination of these primitives are called *strand-displacement systems*,^1^ and can produce complicated and sophisticated reaction networks.

At the sequence level, each domain is assigned a particular nucleotide sequence, and its complement’s sequence is determined by Watson–Crick base pairing. However, in sequence-level analysis and simulation, we allow the full range of binding between any pair of complementary nucleotides, including G-T wobble base pairs. Figure 1*c* shows examples of sequence-level secondary structure, which may not exactly match the intended domain-level structures. Additional unimolecular and bimolecular reactions are also possible at the sequence level (figure 1*d*). Poor sequence design can lead to sequence-level structures or reactions that interfere with the system’s intended domain-level reactions.

### 2.2. Current methods of domain-level system analysis

Domain-level systems involving multiple steps of strand displacement at multiple sites on different complexes can become difficult and error-prone to analyse by hand. By limiting the reaction types allowed at the domain level (see §3.1), it becomes computationally feasible to automatically enumerate all the domain-level reactions possible between a given set of DNA complexes. Such *reaction enumeration* is performed by the software tools Peppercorn [38] and Visual DSD [36,37] and by the methods proposed by Kawamata *et al.* [50,51]. Many reaction enumerators consider only unpseudoknotted complexes, although expanding the range of allowed complexes to include pseudoknots is an active area of research [52]. Here we provide an overview of the approach taken by Peppercorn, but the other reaction enumerators have similar or related concepts.

**Figure 2.**
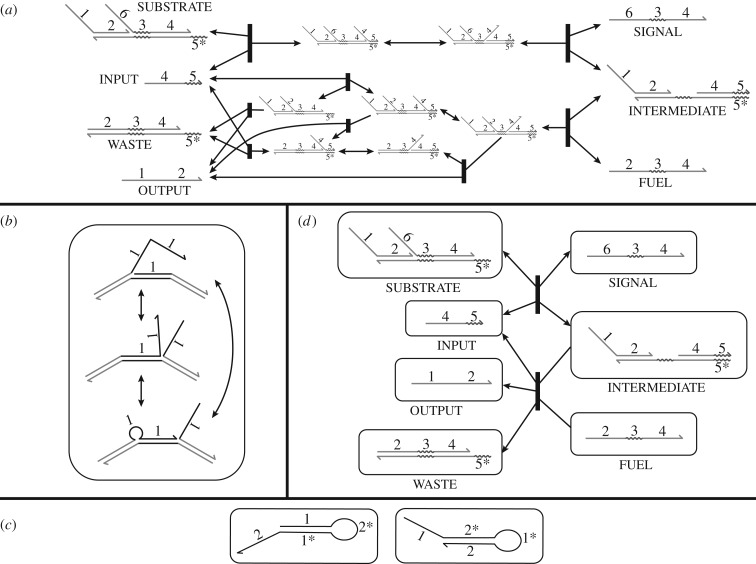
Overview of domain-level system analysis via reaction enumeration. (*a*) An entropy-driven catalytic circuit described by Zhang *et al.* [7], showing the full set of enumerated reactions. Note that dissociation reactions that involve breaking a bound short domain are reversible, while dissociation reactions will be treated as irreversible if completing the strand displacement leaves no exposed toeholds for the reverse reaction. (*b*) Example of a resting macrostate consisting of a complex with three secondary structures that can freely interconvert. Throughout this paper, we use rounded rectangles to indicate resting macrostates of one or more complex conformations. (*c*) Some systems contain distinct domain-level resting macrostates equivalent to the same strand-level complex, but no fast pathways for interconversion. KinDA is not well-suited to analysing these systems. (*d*) Reaction condensation describes system behaviour through reactions between resting macrostates, rather than specific conformations. This change incorporates the separation of timescales assumption, and almost always simplifies the reaction network significantly. Note that the final reaction producing INPUT, OUTPUT and WASTE is shown as irreversible because timescale separation precludes the possibility of trimolecular reactions.

Figure 2*a* shows an example of an entropy-driven catalyst system [7] described at the domain level. This relatively simple system uses six domains to define seven complexes, with additional transient intermediates predicted by reaction enumeration. To simplify the reaction network, one may apply a timescale separation during reaction enumeration, classifying all interactions as either *fast* or *slow*. By default, unimolecular reactions are considered fast and bimolecular reactions slow, while reactions involving three or more molecules do not occur.^2^ Separation of timescales greatly simplifies domain-level analysis of the system and can allow complete enumeration of all reactions in cases where the full network would be too large or infinite. Note that timescale separation according to unimolecular versus bimolecular reactions correctly describes system behaviour in the low concentration limit.

Timescale separation motivates a construct called the *resting macrostate*, a set of conformations that are strongly connected by fast reactions but have no outgoing fast reactions.^3^ Resting macrostates are stable on the timescale of the fast reactions. Examples of resting macrostates are shown in figure 2*b*,*c*.

Detailed reaction enumeration produces an exhaustive set of reactions between one or more complexes in terms of their specific domain-level conformations. *Reaction condensation* creates a new set of reactions by taking the directly enumerated reactions, referred to as the detailed reactions, and combining each slow reaction with a series of subsequent fast reactions into a single reaction. This process is described in more detail by Grun *et al.* [38]. In contrast to detailed reactions, these *condensed reactions* have resting macrostates as reactants and products. Figure 2*d* shows the condensed reactions for the detailed reaction network in figure 2*a*.

The sets of detailed reactions and condensed reactions can be examined to determine if the domain-level system specification is logically correct. In simple systems, this verification can be performed by direct inspection of either set of reactions. In more complex systems, other methods are necessary, such as bisimulation [41], pathway decomposition [40] or serializability analysis [39].

### 2.3. Current methods of sequence-level system verification

While domain-level verification is often a necessary preliminary test of a system, additional verification is required after specific sequences are assigned to each domain. The increased state space and possible molecular interactions at the sequence level make it difficult to directly apply the techniques used at the domain level. In particular, logical proof is much more challenging. This motivates the use of alternative approaches, such as stochastic simulation, for sequence-level verification.

Previous methods for sequence-level analysis do not use the original domain-level specification of a system, instead performing de novo analysis based on the sequence information alone [43–48]. Thermodynamic analysis tools like NUPACK [45], ViennaRNA [44] and the mfold web server [43] analyse the probability of allowed secondary structures assuming the Boltzmann equilibrium has been reached. This analysis is suitable when considering very fast reactions because thermodynamic equilibrium is reached over short timescales and kinetic effects are less significant.

When kinetic considerations become relevant, stochastic simulators may be used to follow conformational changes and reactions as they happen. Stochastic nucleic acid simulators that operate at the nucleotide sequence level, such as Kinfold [46], Kinefold [47] and Multistrand [48], consider reaction kinetics through the space of secondary structures via elementary steps that involve the binding and unbinding of single base pairs. Rate constants for longer reaction pathways can be derived from multiple stochastic trajectories, revealing kinetic properties hidden by thermodynamic analysis.

While it is possible to collect sequence-level data through tools like NUPACK [45] and Multistrand [48], a naive brute-force approach of simulating an entire system is usually too slow and inefficient for anything other than simple DNA strand-displacement systems. A reasonable simplification is to simulate only parts of the system at a time; to make this idea effective, the simulations must be chosen intelligently so that data about the complete system can be inferred from data on its components. In the subsequent sections, we show that domain-level analysis can provide a ‘sketch’ of system behaviour appropriate for this guided analysis.

## 3. Methods

In this paper, we describe a two-part framework for performing probabilistic sequence-level verification based on a comparison between domain- and sequence-level analyses. Reaction enumeration and condensation at the domain level produce a description of the expected resting macrostates and resting macrostate reactions. We can verify the sequence-level system by checking that the enumerated domain-level resting macrostates adopt expected conformations and the condensed reactions occur at appropriate rates. In addition, other unenumerated complexes and reactions must not occur at levels significant enough to affect system function.

The subsections that follow describe this approach in detail. Section 3.1 provides the definitions of the DNA system components used by KinDA. Section 3.2 lists the particular software tools used by KinDA and the relevant features they provide. The remainder of §3 describes in detail how KinDA relates domain- and sequence-level system constructs and how relevant system parameters are estimated via stochastic simulation.

### 3.1. Basic definitions

We consider DNA systems at three levels of granularity: the *sequence level*, where each DNA component is specified with particular nucleotide sequences; the *domain level*, where each DNA component is specified with domains and without regard to nucleotide sequence and the *strand level*, where each DNA component is considered without regard to secondary structure.

**Definition 3.1.**

At the domain and strand levels, a *domain* is defined by an identifier and a positive integer specifying the domain length in nucleotides. At the sequence level, a domain is defined by its identifier and a sequence of bases (*b*_1_, *b*_2_, …, *b*_*n*_), *n* ≥ 1, where each *b*_*i*_ ∈ {*A*, *T*, *C*, *G*}. The sequence of a domain’s complement is determined by Watson–Crick base pairing.

**Definition 3.2.**

A *strand* is defined by an identifier and a sequence of domains (*d*_1_, *d*_2_, …, *d*_*n*_), *n* ≥ 1, ordered from 5′ to 3′ ends.^4^

**Definition 3.3.**

A *secondary structure* or *conformation* describes how a sequence of connected DNA strands are bound to each other. At the domain level, each domain is either completely unbound or completely bound to exactly one complementary domain. At the sequence level, each nucleotide is either unbound or bound to a single complementary nucleotide (i.e. Watson–Crick complement or G-T wobble pair).^5^ At the strand level, secondary structure is not considered.

**Definition 3.4.**

At both the domain and sequence levels, a *complex* is defined by an identifier, a sequence of strands (*s*_1_, *s*_2_, …, *s*_*n*_), *n* ≥ 1, and a secondary structure. At the strand level, a complex is defined by its identifier and strands but lacks a particular secondary structure.^6^

**Definition 3.5.**

A *reaction* is defined by two multisets of complexes, written as

$$A_{1}+A_{2}+\cdots \overset{k}{\to }P_{1}+P_{2}+\cdots$$

or, simply,

$$A\overset{k}{\to }P$$

for reactant multiset $A=\{|A_{1},A_{2},\ldots |\}$, product multiset $P=\{|P_{1},P_{2},\ldots |\}$, and rate constant *k*.

The remainder of this section describes features at the domain and strand levels. Because reaction enumeration is rarely feasible at the sequence level, these features do not apply to sequence-level systems.

At the domain level, reaction enumeration produces reactions of the following types: two complementary unbound domains bind to each other; two bound domains unbind from each other;^7^ one or more unbound domains may each displace an identical nearby bound domain via three-way branch migration, or pairs of bound domains may exchange partners with nearby identical pairs via four-way branch migration.

**Definition 3.6.**

The *detailed reactions* of a domain-level system are all reactions predicted by reaction enumeration. These may involve complexes not explicitly specified in the system description, if these complexes were predicted by reaction enumeration. Bimolecular reactions are classified as *slow* and unimolecular reactions may be classified by the enumerator as either *fast* or *slow*. The strand-level detailed reactions are found by converting all reactions to strand-level equivalents and removing those whose reactants and products are equal.

**Definition 3.7.**

A domain-level *resting macrostate* or *resting set* is a set of domain-level complexes strongly connected by fast reactions with no outgoing fast reactions. A *resting complex* refers to any complex within some resting macrostate. Any other complex is termed a *transient complex*. At the strand level, a resting macrostate contains only a single strand-level complex.

A resting macrostate will always be stable on the timescale of the fast reactions, with each constituent resting complex having an equilibrium relative concentration. By contrast, transient complexes have at least one outgoing fast reaction that leads toward a resting macrostate, and thus they will vanish quickly via one of these reactions. Note that all complexes within a resting macrostate will have the same set of strands, in the same order, so it is sometimes instructive to refer to a resting macrostate as a set of secondary structures over the same strands. In fact, in cases of interest for analysis by KinDA, there is at most one resting macrostate per strand-level complex.

**Definition 3.8.**

The *reaction subnetwork* for multiset of resting macrostates $A=\{|A_{1},A_{2},\ldots |\}$ is the subset of detailed reactions consisting of the slow reactions possible with the members of A and the fast reactions possible after any such slow reaction or subsequent fast reactions. Throughout this paper, A contains one or two resting macrostates.

**Definition 3.9.**

The *condensed reactions* or *resting macrostate reactions* are the reactions produced by reaction condensation (see §2.2 and [38]). Each condensed reaction has reactant and product multisets consisting of resting macrostates rather than complexes.

### 3.2. Software dependencies

Our methods rely on three types of analyses: reaction enumeration at the domain level and thermodynamic and kinetic analyses at the sequence level. Although KinDA currently relies on the following three packages, it is reasonable to expect that our framework could be adapted to use any tool satisfying a few basic assumptions.

For domain-level reaction enumeration, we use the Peppercorn enumerator [38] because it considers a general, widely used class of DNA complexes—arbitrary, non-pseudoknotted, multistranded complexes—and it provides both a detailed and a condensed reaction network. We anticipate that the KinDA framework could be used with other enumerators so long as the detailed reactions consist of slow bimolecular reactions and fast and slow unimolecular reactions.

For sequence-level thermodynamic analysis, we use the Nucleic Acids Package (NUPACK) [45]. NUPACK allows the sampling of arbitrary unpseudoknotted secondary structures from the equilibrium Boltzmann distribution of conformations possible for a given strand-level complex. This capability is used to estimate the probability of a resting macrostate being well formed.

For sequence-level kinetic analysis, we use Multistrand [48] to produce stochastic elementary step simulations of reaction trajectories between DNA complexes. Multistrand provides a special simulation mode called ‘First Step Mode’ (FSM). FSM simulations break the reaction trajectory into two parts: the initial binding step and the folding trajectory that follows, with any particular simulation containing separate data on both steps. The initial binding step occurs between a pair of unbound nucleotides that have the potential to form a base pair, one from each of the initial molecules, whose states are Boltzmann sampled. The rate of this step is estimated from the number of different such pairs that could form in the initial state. The subsequent folding trajectory step is simulated until any of a set of predetermined stop states has been reached; stop conditions are specified as a set of sequence-level complexes that must be present. This mode is well suited to simulations at low concentrations, when separate complexes will adopt their equilibrium Boltzmann distributions prior to interacting with each other.

### 3.3. Relating domain-level and sequence-level resting macrostates and secondary structure

**Figure 3.**
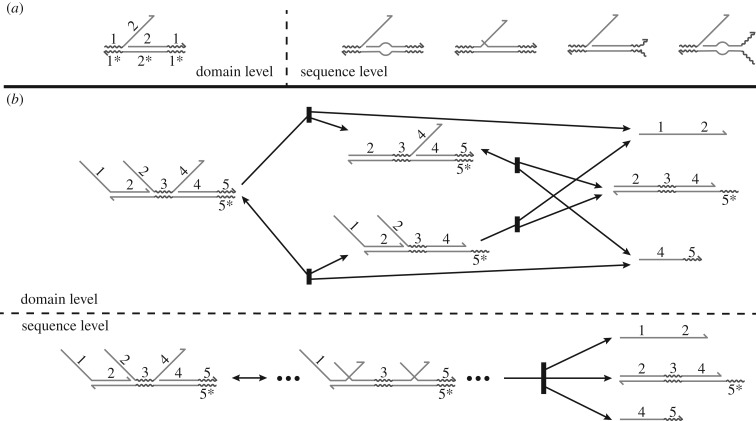
Sequence-level secondary structures and reaction pathways do not directly correspond to domain-level secondary structures. Characterizing the domain-level constructs based on sequence-level data requires mapping between the two. (*a*) Sequence-level conformations different from a domain-level conformation may or may not be functionally equivalent. (*b*) In the entropy-driven catalyst described by Zhang *et al.* [7], this domain-level reaction pathway assumes that the branch migration reactions on the left (domain 2) and the right (domain 4) occur sequentially, with one completing before the other begins. Simulating these reactants at the sequence level commonly produces trajectories in which both domains undergo branch migration simultaneously, so many of these trajectories do not correspond to any specific domain-level reaction pathway. This motivates our approach of only considering strand-level complexes during stochastic simulation when identifying spurious trajectories.

Sequence-level interactions may not have direct counterparts in the domain-level system. For instance, sequence-level conformations may differ from domain-level conformations in ways that may or may not change the behaviour of the complex (figure 3*a*). Similarly, sequence-level and domain-level reaction trajectories may differ even when no undesired behaviour occurs. For instance, as in figure 3*b*, simultaneous branch migration on different parts of a complex will produce sequence-level trajectories with intermediates quite different from domain-level predictions. In this and the following section, we develop a precise relationship between secondary structures and reaction pathways at the sequence and domain levels.

To determine whether a sequence-level complex belongs to a domain-level resting macrostate, we first assume that no two distinct domain-level resting macrostates share the same ordered list of strands up to circular permutation. At the sequence level, this is equivalent to assuming that there are no significant kinetic barriers between different low-energy conformations of the corresponding strand-level complex. This assumption implies that the sequence-level conformations observed on a complex composed of these strands will follow the equilibrium Boltzmann distribution. Although many DNA systems satisfy this assumption, those that do not (e.g. figure 2*c*) should be analysed by this framework with caution.

Of particular interest is the probability that a sequence-level complex will adopt a conformation similar to expected domain-level complexes. To make this notion precise, we associate each domain-level conformation with a set of functionally similar sequence-level conformations. The following definitions are motivated by the fact that the domains in a complex represent the smallest functional units of the molecule. If all domains in a sequence-level secondary structure are bound in approximately the same manner as a domain-level secondary structure, then it is reasonable to expect that a sequence-level complex with that conformation will function similar to the domain-level complex.

**Definition 3.10.**

A sequence-level secondary structure *T*_s_ is a *p-approximation* of domain-level secondary structure *T*_d_ if they share the same ordered strands (up to circular permutation) and, for every domain in each strand, the fraction of nucleotides in *T*_s_ that are unbound or bound to the same targets as in *T*_d_ is greater than or equal to *p*, which is a fraction between 0 and 1.

**Definition 3.11.**

A sequence-level secondary structure *T*_s_ is *p-spurious* if it is not a *p*-approximation for any domain-level secondary structure in the domain-level system specification. Otherwise, we say *T*_s_ is *well-formed* when the value of *p* is clear from context.

**Figure 4.**
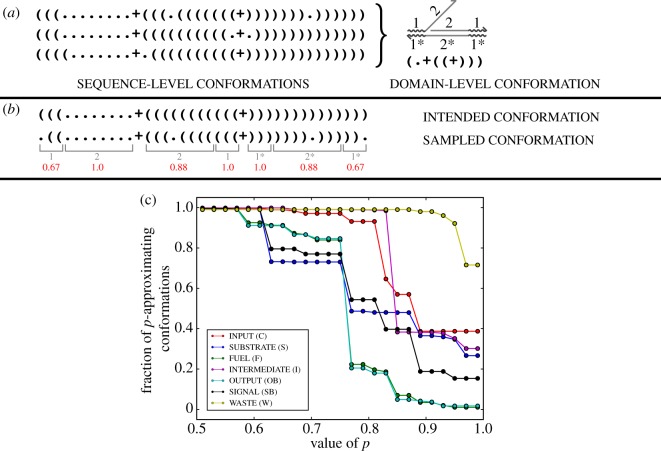
Correspondence between sequence-level and domain-level secondary structure. (*a*) Examples of sequence-level conformations that are *p*-approximations of a domain-level conformation. This example uses a value of *p* = 0.8 so that each instance of domain 2, which is eight nucleotides long, may have at most one nucleotide incorrectly bound. Note that the toehold domain 1 has only three nucleotides, so it may not have any nucleotides incorrectly bound with this value of *p*. (*b*) To determine whether a secondary structure is a *p*-approximation, we calculate the fraction of correctly bound nucleotides (red text) in each domain. To be considered a *p*-approximation, this fraction must be greater than or equal to *p* for every domain. In this case, the sampled structure would be a *p*-approximation for any $p\leq \frac{2}{3}$. (*c*) Effect of *p* on the probability that a sampled sequence-level conformation will be well formed, for all resting macrostates in the entropy-driven catalyst system (figure 2*d*) for the experimental DNA sequences, at a temperature of 25°C and $[{\text{Na}}^{+}]=1\;\text{M}$ (cf. figure 7).

Figure 4*a*,*b* shows the application of definition 3.10 to particular sequence-level secondary structures. Note that the value of *p* is specific to the particular system and application, and the user is responsible for choosing a value of *p* that accounts for the sensitivity of the resting macrostate to non-ideal domain behaviour. Using *p* > 0.5 is recommended, as in that case a given sequence-level complex can be a *p*-approximation of at most one domain-level resting macrostate. As a general rule, a reasonable *p* may be 0.51 to ensure that three-way branch migration domains, which will often exhibit partial migration, are not classified as spurious. If leak reactions are of particular concern, a higher *p* may be necessary to recognize the opening of single base pairs in a double-stranded region.

Figure 4*c* demonstrates the effect of *p* on the probability that a sampled secondary structure will not be *p*-spurious for the resting macrostates in the entropy-driven catalyst system (figure 2*d*). Increases in *p* lower the probability of being well formed because higher *p* represents a more restrictive condition on approximating a domain-level conformation. This system, which lacks active branch migration domains in the resting macrostates, retains reasonably well-formed resting macrostates for *p* ≤ 0.77. The process of computing these probabilities is described in §3.5.1.

### 3.4. Relating domain-level and sequence-level reaction pathways and reaction rates

When designing and analysing sequence-level reaction pathways, we consider the following augmented model for the interactions between one or two resting macrostates, building on the approach developed in [48]:

**Definition 3.12.**

The *first-step model* for multiset of resting macrostates A is the set of all reaction pairs $R_{i}$ of the form

$$R_{i}\;:\;A\overset{k_{1}^{i}}{⟶}A_{i}\overset{k_{2}^{i}}{⟶}P_{i},$$

where each $P_{i}=\{|P_{1}^{i},P_{2}^{i},\ldots |\}$ is the *i*th multiset of possible final product resting macrostates resulting from a domain-level reaction pathway beginning with A. The reaction pair with $P_{0}=A$ is termed the *unproductive reaction* and is always included if A has two or more reactants.^8^ Reactions with $P_{i}$, *i* = 1, 2, … , are termed *productive reactions*. In addition, a *spurious reaction* $R_{s}$ is included of the form

$$R_{s}\;:\;A\overset{k_{1}^{s}}{⟶}A_{s}\overset{k_{2}^{s}}{⟶}P_{s}.$$

For brevity, we often refer to reaction pairs in shorthand as a single reaction^9^

R : A→P.

When estimating rate constants or performing standard mass-action chemical kinetics simulations, the two steps are considered separately.

The first-step model separates each reaction into two steps. For bimolecular reactions, these can be intuitively understood as modelling an initial bimolecular interaction followed by a unimolecular reconfiguration, which allows both the reaction rate’s concentration dependence and the reaction’s temporal extent to be explicitly modelled. For unimolecular reactions, the $k_{1}^{i}$ and $k_{2}^{i}$ determine the rate of initiating the reaction and how long it takes to complete, respectively. The intermediates $A_{i}$ represent a coarse-graining of trajectories through intermediate complexes based on the final product set $P_{i}$ they are destined to reach. They do not refer to particular complexes or macrostates themselves. See [48] for a discussion of this treatment of $A_{i}$ and its implications.

**Figure 5.**
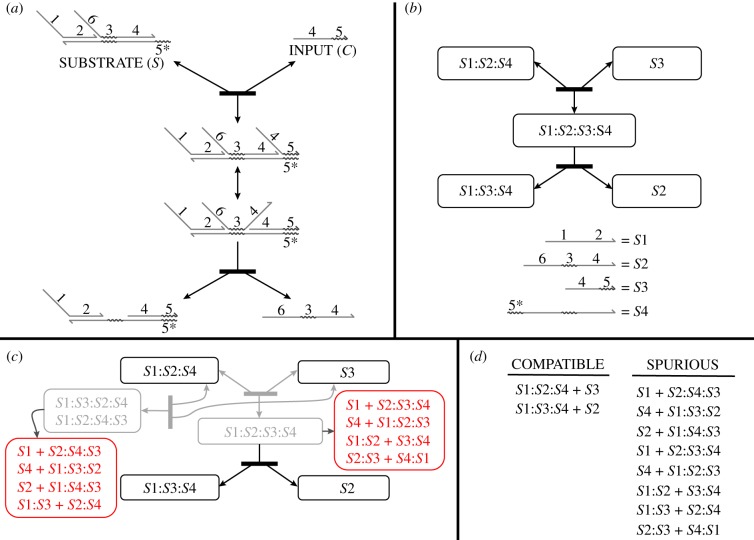
Automatic determination of stop states between two resting macrostates for the entropy-driven catalyst described by Zhang *et al.* [7]. (*a*) The domain-level reaction subnetwork between resting macrostates *S* and *C*. Note that these resting macrostates each have only one conformation, and that the final reaction is shown as irreversible because the reverse reaction is not part of this reaction subnetwork. (*b*) Strand-level reaction subnetwork between resting macrostates *S* and *C*. The strands are labelled *S*1, *S*2, *S*3 and *S*4. Observe that the two intermediate domain-level complexes are conflated into a single strand-level complex because these complexes differ only in secondary structure and not in strand order. (*c*) The spurious stop states are automatically determined by accounting for improper dissociation after some bimolecular binding step. For every predicted strand-level state of the simulation box, each strand-level complex after the initial binding event is considered as a candidate (grey) for improper dissociation. A dissociation event partitions the ordered strands of a complex into two separate lists, and all such partitions that lead to strand-level states not reachable via enumerated strand-level pathways are included as spurious stop states (red). Note that improper binding producing an unenumerated strand-level complex is not considered spurious unless the complex dissociates into an unenumerated form. If the complex dissociates into the original reactants, this is instead classified as unproductive. For unimolecular spurious stop states, no initial binding step is considered, and all dissociation events producing unexpected strand-level simulation states are included as spurious stop states. (*d*) Final list of compatible and spurious stop states.

When analysing the reactions of the first-step model of A, we consider simulated trajectories beginning with a single copy of each element of A. While any simulated trajectory will, given enough time, reach one of the expected product states $P_{i}$, it is important to identify when a simulated trajectory deviates significantly from the expected enumerated reaction pathways. Such trajectories should correspond to the spurious reaction $R_{s}$ rather than any of the $R_{i}$. To understand the difficulty of determining this deviation, consider the reaction in the Zhang *et al.* system [7] shown in figure 3*b*. Existing domain-level reaction enumerators will predict the branch migration of each domain to happen sequentially, with the branch migration completing on one side before beginning on the other. However, at the sequence level, these branch migrations usually happen simultaneously, so that a well-behaved simulated trajectory will not directly match any domain-level reaction pathways to the final state. For this reason, we instead use the strand-level reaction subnetwork, which provides a level of detail intermediate between the domain-level subnetwork and the condensed reactions.^10^ Figure 5*a*,*b* shows a domain-level reaction subnetwork and the corresponding strand-level reaction subnetwork.

**Definition 3.13.**

At the sequence level, a reaction trajectory beginning with multiset of resting macrostates A is *spurious* if it forms a multiset of strand-level complexes not possible by following reactions in the strand-level reaction subnetwork before reaching one of the non-spurious product multisets $P_{i}$. Otherwise, the trajectory is *compatible*.

**Definition 3.13**

classifies a reaction trajectory based on each observed strand-level ‘state of the simulation box’ (i.e. the multiset of strand-level complexes in a reaction trajectory at a given time point). Observing a strand-level state not reachable via the strand-level reaction subnetwork indicates that an unexpected dissociation event has occurred.^11^ We consider these trajectories spurious even if the trajectory were to later rejoin an expected reaction pathway, because they do not correspond to the behaviour predicted at the domain level. For this reason, these trajectories are undesirable from the perspective of confirming that the system’s behaviour matches domain-level predictions.

Each stochastic simulation halts as soon as the trajectory can be classified as either spurious or compatible, as identified by the methods discussed below. The complexes in the initial and non-spurious final states of compatible trajectories have a direct correspondence to resting macrostates in the domain-level model. The following simulation modes allow the user to adjust how closely each sequence-level complex must resemble its corresponding resting macrostate.

**Definition 3.14.**

In *ordered-complex* mode, simulations use only the strand-level reactants and products to determine initial and final states of the reaction trajectory. Initial states are sampled from the Boltzmann distribution of secondary structures possible for the given strand-level reactant and the simulation halts as soon as the strand-level elements of some $P_{i}$ are produced. In *count-by-domain* mode, the initial states are sampled from the Boltzmann distribution of conformations for the same strand-level complex, with the condition that the conformation is a *p*-approximation of one of the domain-level conformations. Simulations halt when the product secondary structures satisfy this same condition. In *count-by-complex* mode, the initial and final states are similarly restricted but with the fractional defect computed over the entire complex, rather than for each domain.

In most cases, ordered-complex mode is sufficient to achieve good rate estimates. The additional modes are slower to simulate because they require more involved checking of the system state at every time step, so are only recommended when necessary. In particular, count-by-complex mode is provided as a less accurate version of count-by-domain mode to reduce compute times. Note that the initial and final states of a trajectory may be configured with different modes. The implications of these three modes are explored in the Groves *et al*. case study (§4.4).

To identify spurious trajectories, KinDA automatically determines a minimal set of strand-level states as ‘stop states’ for the stochastic simulator. Figure 5*c*,*d* shows the process of determining the stop states for a selected reaction from the Zhang *et al.* system [7]. In each spurious stop state, the complexes have no relationship to domain-level resting macrostates, so the modes in definition 3.14 do not apply. These halting conditions effectively always apply ordered-complex mode.

Note that although the spurious first-step model product $P_{s}$ conflates all possible product multisets formed by a spurious trajectory, the particular unexpected strand-level complexes formed, as well as separate *k*_1_ and *k*_2_ rate constants for their formation, are available to the user to help debug the reason for their occurrence, for instance using domain-level-agnostic tools such as NUPACK or Multistrand. Because these complexes lack a domain-level description of their behaviour, KinDA is not well equipped to characterize their properties directly.

The following definition describes the correspondence between simulated reaction trajectories beginning with A and the reactions of the first-step model for A. Note that each trajectory corresponds to at most one first-step model reaction.^12^

**Definition 3.15.**

Consider non-spurious reaction $R_{i}$ in the first-step model for A, with final products $P_{i}$. A compatible reaction trajectory *corresponds* to $R_{i}$ if the trajectory begins with A and ends with $P_{i}$. A spurious reaction trajectory corresponds to $R_{s}$, the spurious reaction.

A complete characterization of the first-step model reactions includes estimates for the rate constants $k_{1}^{i}$ and $k_{2}^{i}$ (see §3.5). The full set of productive, unproductive, and spurious reactions and their rate constants, which we may call the *first-step CRN*, are intended to be suitable for simulation by any off-the-shelf CRN simulator (e.g. [53]; in this paper, we use the simulator provided with the Nuskell compiler [42]) according to standard mass-action chemical kinetics using either discrete stochastic (Gillespie, continuous-time Markov chain) semantics [54] or continuous deterministic (ordinary differential equation, ODE) semantics [55]. In this way, simulations of the first-step CRN allow us to examine the predicted behaviour of the system when more than one copy of each species may be present, or when given concentrations of each species are specified. Note that although every reaction in the first-step CRN has a non-zero probability of occurring as a Multistrand simulation trajectory, the probability may be extraordinarily small—so when an insufficient number of stochastic simulations in Multistrand are performed, no such trajectories may be observed. In this case, KinDA’s rate constant estimate are informed only by the number of attempted trials and may be extreme overestimates; thus, reactions with no observed corresponding trajectories may (and perhaps should) be omitted from simulations.

### 3.5. Estimating system parameters

This section describes how, after sequence assignment, the parameters of the sequence-level system can be estimated from simulation data to determine if the system behaviour will match domain-level predictions. We estimate two features of the sequence-level system:

(1) For each domain-level resting macrostate, the *conformation probabilities* (i.e. the likelihood that a corresponding sequence-level structure will adopt a *p*-approximation of each domain-level conformation, for a user-provided value of *p*).
(2) For each first-step model reaction, the reaction rates *k*_1_ and *k*_2_ (using no extra parameters for ordered-complex mode, but using a user-provided value *p*′ for count-by-domain and count-by-complex modes).

For each parameter, KinDA’s user interface allows the user to specify a desired precision of the result, and sampling or simulations are performed as needed to achieve that result; as a consequence, KinDA requires useable error estimates even in the early stages before any successful cases have been observed, and when few have been observed.

#### 3.5.1. Estimating conformation probabilities

Recall our assumption that the sequence-level conformations adopted by the strands in a resting macrostate follow the equilibrium Boltzmann distribution (§3.3). Given a resting macrostate and its predicted domain-level conformations, we apply this assumption to estimate the probability of a sequence-level secondary structure being a *p*-approximation of each predicted conformation and of being spurious.

Using dynamic programming, the probability of adopting any sequence-level conformation satisfying certain constraints can be computed explicitly in *O*(*n*^3^) time, where these constraints take the form of particular base pairs being bound or unbound, while other base pairs are allowed to vary [56]. However, the definition of a *p*-approximation describes a different type of constraint not covered by this algorithm. Instead, we estimate conformation probabilities by empirically sampling sequence-level secondary structures from the Boltzmann distribution using NUPACK [45]. Each secondary structure in a set of samples can be classified as *p*-spurious or a *p*-approximation of at least one of the expected domain-level secondary structures. Note that if *p* ≤ 0.5, a particular sampled sequence-level secondary structure may match multiple domain-level secondary structures, so we will always use *p* > 0.5.

Let *N* denote the total number of samples collected and *N*_*i*_ denote the number of samples that are a *p*-approximation of the *i*th domain-level conformation. *p*_*i*_ is the true probability of the *i*th conformation, and ${\hat{\;p}}_{i}$ is our estimate of this probability. We use *i* = s to refer to the corresponding values for the spurious conformation. Note that for a given *N* and *i*, *N*_*i*_ is a binomial random variable $N_{i}∼\mathrm{binomial}(N,p_{i})$.

##### 3.5.1.1. Estimation of *p*_*i*_

A naive approach to estimating *p*_*i*_ might be to calculate the maximum-likelihood estimate (MLE) for the probability; from basic statistics, this estimate is ${\hat{\;p}}_{i}^{\mathrm{MLE}}=N_{i}/N$. However, this approximation may be misleading: for example, when *N*_*i*_ = 0 we get ${\hat{\;p}}_{i}^{\mathrm{MLE}}=0$. That is, the conformation probability is estimated equal to zero, despite the fact that the secondary structure may clearly be possible. As we will show, this situation also makes it difficult to determine the error on the estimate.

Instead, we use the Bayesian estimate of the expectation of the conformation probability given *N* and *N*_*i*_. Using a uniform prior distribution on *p*_*i*_, the expectation is exactly

3.1 $${\hat{\;p}}_{i}=E[p_{i}|\text{data}]=\frac{N_{i}+1}{N+2}.$$

##### 3.5.1.2. Error estimation for *p*_*i*_

Error estimation using maximum-likelihood methods may also be misleading. The maximum-likelihood estimate is

$${\hat{\sigma }}_{\;p_{i}}^{\mathrm{MLE}}=\sqrt{\frac{{\hat{\;p}}_{i}^{\mathrm{MLE}}(1-{\hat{\;p}}_{i}^{\mathrm{MLE}})}{N}}.$$

When *N*_*i*_ = 0 or 1, ${\hat{\sigma }}_{\;p_{i}}^{\mathrm{MLE}}=0$, which is clearly inaccurate. Without a more suitable error estimate, we cannot judge our confidence in the result or determine whether additional samples should be drawn.

We instead measure the spread in the possible values of *p*_*i*_ with the standard deviation of its posterior distribution given *N* and *N*_*i*_, calculated using Bayesian inference

3.2 $${\hat{\sigma }}_{\;p_{i}}=\sqrt{\frac{{\hat{\;p}}_{i}(1-{\hat{\;p}}_{i})}{N+3}}.$$

Derivations for equations (3.1) and (3.2) can be found in electronic supplementary material, appendices A.1 and A.2.

#### 3.5.2. Estimating reaction rates (bimolecular reactions)

In the paragraphs that follow, it is helpful to note that a given Multistrand simulated trajectory is not representative of a trajectory sampled from all collisions that would occur in a test tube. Multistrand FSM trajectories are reactions between single copies of *A* and *B* with initial states of *A* and *B* chosen from the Boltzmann distribution of possible conformations of each macrostate, with the first step of the trajectory being a bimolecular interaction forming a base pair between *A* and *B*. The distribution over trajectories sampled this way is referred to in the rest of this discussion as the FSM distribution. By contrast, a trajectory sampled from the distribution of all test-tube collisions is consistent with the chemical master equation (CME), and will be weighted by an associated rate constant. This distribution is referred to as the CME distribution. Expectations taken over one or the other distribution may differ; where ambiguous, we will specify which of these distributions we are using.

Consider the interactions between any two resting macrostates *A* and *B*. Each simulated reaction trajectory between *A* and *B* corresponds to a single reaction in the first-step model, except when the sampled conformations do not allow an immediate bimolecular step. Multistrand reports two values for each trajectory that are of use to us: *k*_coll_, the rate constant for the bimolecular collision between the sampled conformations of *A* and of *B*; and *τ*_2_, the time taken to complete the unimolecular step [48]. Here, we generalize methods from [48] to combine these observations into a single estimate of $k_{1}^{i}$ and $k_{2}^{i}$ for each reaction

$$R_{i}\;:\;A+B\overset{k_{1}^{i}}{⟶}AB_{i}\overset{k_{2}^{i}}{⟶}P_{i}.$$

For the following discussion, *N* denotes the total number of simulated FSM trajectories between *A* and *B*, and *N*_*i*_ denotes the number of these trajectories corresponding to reaction $R_{i}$. The *N* trajectories are indexed with a variable *n* = 1, …, *N*. Each trajectory is characterized by the binary values $S_{i}^{n}$, which is 1 if and only if the *n*th trajectory corresponds to reaction $R_{i}$, and $k_{\mathrm{coll}}^{n}$ and $\tau_{2}^{n}$, which are the values reported by Multistrand for the *n*th trajectory. Trajectories with no initial step have all $S_{i}^{n}=0$.

##### 3.5.2.1. Estimation of *k*_1_

For reaction $R_{i}$, we estimate $k_{1}^{i}$ using a Bayesian approach. $k_{1}^{i}$ is defined as the rate constant for collisions between *A* and *B* in a test tube that ultimately lead to products $P_{i}$. This is equivalent to the following:

3.3 $$k_{1}^{i}=p_{i}k_{\mathrm{coll},i}=E[S_{i}^{n}]*k_{\mathrm{coll},i}=E[S_{i}^{n}k_{\mathrm{coll}}^{n}],$$

where $p_{i}=E[S_{i}^{n}]$ is the probability that a trajectory sampled from the FSM distribution will have $S_{i}^{n}=1$ and *k*_coll,*i*_ is the expectation of $k_{\mathrm{coll}}^{n}$ taken over only these trajectories with $S_{i}^{n}=1$.

Using the expectation of $k_{1}^{i}$ given the data as our estimate, we have the following formula for ${\hat{k}}_{1}^{i}$:

3.4 $${\hat{k}}_{1}^{i}=E[k_{1}^{i}|\text{data}]=\frac{\sum_{S_{i}^{n}=1}k_{\mathrm{coll}}^{n}}{N+2},$$

where to simplify the calculation we make the assumption that *p*_*i*_ and *k*_coll,*i*_ are independent random variables, with *p*_*i*_ having a uniform prior on [0, 1] and *k*_coll,*i*_ having prior $P(k_{\mathrm{coll},i})\propto 1/{\left(k_{\mathrm{coll},i}\right)}^{3}$.

##### 3.5.2.2. Error estimation for *k*_1_

We estimate the error on $k_{1}^{i}$ with the following equation for the standard deviation of the posterior distribution of $k_{1}^{i}$ given the observed trajectories:

3.5 $${\hat{\sigma }}_{k_{1}^{i}}={\hat{k}}_{1}^{i}\sqrt{\frac{2N-N_{i}+1}{N_{i}(N+3)}}.$$

##### 3.5.2.3. Estimation of *k*_2_

When estimating $k_{2}^{i}$ for reaction $R_{i}$, we make the simplifying assumption that the unimolecular step times $\tau_{2}^{n}$ for reaction trajectories corresponding to $R_{i}$ are drawn from a distribution with mean $1/k_{2}^{i}$, where this mean is taken over trajectories following the CME distribution. We use the following estimator for $k_{2}^{i}$:

3.6 $${\hat{k}}_{2}^{i}=\frac{\sum_{S_{i}^{n}=1}k_{\mathrm{coll}}^{n}}{\sum_{S_{i}^{n}=1}k_{\mathrm{coll}}^{n}\tau_{2}^{n}}.$$

##### 3.5.2.4. Error estimation for *k*_2_

The standard deviation of the expected unimolecular reaction time *τ*_2,*i*_ is calculated using equation (3.6), above, which represents a weighted sum of the simulated reaction times $\tau_{2}^{n}$ over successful trajectories. Using the inversely proportional relationship between $k_{2}^{i}$ and the *τ*_2,*i*_, we can derive an estimate for the standard deviation of the estimate for $k_{2}^{i}$ to be

3.7 $${\hat{\sigma }}_{k_{2}^{i}}={\left({\hat{k}}_{2}^{i}\right)}^{2}\sqrt{\frac{\sum_{S_{i}^{n}=1}k_{\mathrm{coll}}^{n}{\left(\tau_{2}^{n}-\frac{1}{{\hat{k}}_{2}^{i}}\right)}^{2}}{(N_{i,\mathrm{eff}}-1)\sum_{S_{i}^{n}=1}k_{\mathrm{coll}}^{n}}},$$

where

$$N_{i,\mathrm{eff}}=\frac{{\left(\sum_{S_{i}^{n}=1}k_{\mathrm{coll}}^{n}\right)}^{2}}{\sum_{S_{i}^{n}=1}{\left(k_{\mathrm{coll}}^{n}\right)}^{2}}.$$

Derivations for equations (3.4) and (3.5) are found in electronic supplementary material, appendices A.3 and A.4, respectively. Equation (3.6) is generalized from the derivation in [48] for reactants with a single productive reaction. Equation (3.7) is derived in electronic supplementary material, appendix A.5.

#### 3.5.3. Estimating reaction rates (unimolecular reactions)

When slow unimolecular reactions are enumerated at the domain level, the first-step model treats such reactions as two-step reaction pathways with *k*_1_ and *k*_2_. For these reactions, KinDA uses *k*_1_ to represent the probability of the reactant following a particular pathway and *k*_2_ to determine the time taken along the pathway. Multistrand simulations for unimolecular first-step model reactions do not use FSM. The following paragraphs consider first-step model reaction $R_{i}$ for resting macrostate *A*. Trajectories are indexed by *n* = 1, …, *N* and each has an associated trajectory time $\tau_{2}^{n}$.

##### 3.5.3.1. Estimation of *k*_1_

When the first-step CRN is treated as a Markov chain, the probability that resting macrostate *A* will produce $P_{i}$ is

$$P_{A}(i)=\frac{k_{1}^{i}}{\sum_{j}k_{1}^{j}}$$

where *j* ∈ {s, 1, … }. For $k_{1}^{i}\gg k_{2}^{i}$, the rate constant for the overall reaction $A\to P_{i}$ is simply $k_{2}^{i}$. KinDA estimates $k_{1}^{i}$ by attempting to enforce these two constraints. *P*_*A*_(*i*) is estimated with equation (3.1), where *N*_*i*_ is the number of simulated trajectories corresponding to $R_{i}$.

$$k_{1}^{i}=k_{\mathrm{fast}}{\hat{P}}_{A}(i),$$

where $k_{\mathrm{fast}}=k_{\mathrm{scale}}\times \max_{i}\{k_{2}^{i}\}$ enforces that $k_{1}^{i}\gg k_{2}^{i}$ while maintaining the relative values of all *k*_1_ in the first-step model for *A*. Any *k*_scale_ may be used as long as it is large enough that the time taken to generate $P_{i}$ is dominated by the second step.^13^

##### 3.5.3.2. Error estimation for *k*_1_

Because the scale of $k_{1}^{i}$ is not meaningful, we consider only the error in ${\hat{P}}_{A}(i)$, and report

$${\hat{\sigma }}_{k_{1}^{i}}=k_{\mathrm{fast}}{\hat{\sigma }}_{P_{A}(i)},$$

with *k*_fast_ defined as before and ${\hat{\sigma }}_{P_{A}(i)}$ defined as in equation (3.2).

##### 3.5.3.3. Estimation of *k*_2_

Because we guarantee that $k_{1}^{i}\gg k_{2}^{i}$, the time taken to produce $P_{i}$ is determined only by $k_{2}^{i}$. The average time to completion is inversely proportional to the rate constant, so we have

$${\hat{k}}_{2}^{i}=\frac{1}{\tau_{2,i}}$$

where *τ*_2,*i*_ is the mean reaction time for reaction trajectories corresponding to $R_{i}$.

##### 3.5.3.4. Error estimation for *k*_2_

Following identical reasoning as for error estimation of *k*_2_ in the bimolecular case, we have

$${\hat{\sigma }}_{k_{2}^{i}}={\left({\hat{k}}_{2}^{i}\right)}^{2}\;*\;{\hat{\sigma }}_{\tau_{2,i}}={\left({\hat{k}}_{2}^{i}\right)}^{2}\sqrt{\frac{\sum_{S_{i}^{n}=1}{\left(\tau_{2}^{n}-1/{\hat{k}}_{2}^{i}\right)}^{2}}{N_{i}(N_{i}-1)}},$$

where $S_{i}^{n}=1$ if and only if the *n*th trajectory corresponds to $R_{i}$.

### 3.6. Usage and interpretation of the analysis framework

The framework described in this paper can be used to judge the sequences for a single component of a DNA circuit or for the circuit as a whole. For instance, if the interactions between a particular pair of resting macrostates has been previously found to be problematic, KinDA can analyse just these interactions in isolation of the rest of the system with multiple potential sequences to determine which sequences are most probable to produce a functioning system. Once sequences are chosen, the sequence-level system can be verified in its complete form by estimating reaction rates for each reaction in the model.

Given reaction rate estimates, the behaviour of the system can be judged by simulating standard mass-action chemical kinetics, i.e. constructing mass-action differential equations from the first-step model reactions and applying standard numerical ODE solvers. Alternatively, KinDA computes scoring metrics for certain components of the system, as well as for the system overall.

For each resting macrostate in a system, KinDA can compute a bound on the *temporary depletion* of this resting macrostate due to time potentially spent undergoing unproductive reactions. This metric is computed at three levels of detail. Each metric assumes a user-provided maximum concentration *c*_*A*_ for every resting macrostate *A* and provides an upper bound on the temporary depletion assuming concentrations are fixed at this level. For two resting macrostates *A* and *B*, we bound the temporary depletion *A* due to the unproductive reaction between *A* and *B* with *α*_*AB*_

3.8 $$\alpha_{AB}=\frac{K_{AB}c_{B}}{1+\sum_{A^{\prime }}K_{AA^{\prime }}c_{A^{\prime }}},$$

where $K_{AB}=k_{1}^{0}/k_{2}^{0}$ is the association constant for the unproductive reaction between *A* and *B*, based on the rate constant estimates from Multistrand FSM simulations. We similarly bound the total temporary depletion of *A* due to all unproductive reactions involving *A* with *α*_*A*_

3.9 $$\alpha_{A}=\frac{\sum_{A^{\prime }}K_{AA^{\prime }}c_{A^{\prime }}}{1+\sum_{A^{\prime }}K_{AA^{\prime }}c_{A^{\prime }}}.$$

The system-level unproductive reaction score *α* is the maximum temporary depletion of any *A*

3.10 $$\alpha =\max_{A}\{\alpha_{A}\}.$$

Note that these equations implicitly assume that unproductive reactions are on a faster timescale than productive reactions. While systems can be constructed for which this is not true, this assumption generally holds in practice because unproductive reactions tend to consist of weak binding of mismatched sequences and temporary binding by short toeholds, whereas productive reactions involve additional branch migration steps. Because these equations compute the depletion amount due to a single set of maximum concentrations for each reactant, they provide an upper bound on the level of depletion. While true depletion levels will vary from the reported bounds, the total depletion levels should remain below these bounds. In addition, because these estimates are sensitive to the supplied maximum concentrations, circuits for which maximum concentrations cannot be found should not be judged by these scores.

KinDA also computes the *permanent depletion* of a resting macrostate due to spurious reactions. Because the behaviour of spurious products is beyond the scope of the domain-level model and therefore considered unknown, we assume a resting macrostate undergoing a spurious reaction becomes permanently unusable. The fractional depletion rate of a resting macrostate *A* due to a spurious reaction *R*_s_ with resting macrostate *B* is bounded by $c_{B}k_{1}^{s}$. The fractional depletion rate of resting macrostate *A* due to all spurious reactions is bounded (with some abuse of notation) by

3.11 $$\beta_{A}=\sum_{A^{\prime }}c_{A^{\prime }}k_{1}^{s},$$

where each $k_{1}^{s}$ is the bimolecular rate constant of the spurious reaction between *A* and the relevant *A*′. The system-level spurious reaction score is the maximum fractional depletion rate of any resting macrostate *A*, or

3.12 $$\beta =\max_{A}\{\beta_{A}\}.$$

If the user has desired parameter ranges for each productive reaction rate in the system, these can also be used to manually determine if the sequence-level system is well behaved. Because this involves additional knowledge about the expected system behaviour, KinDA does not automatically score this aspect of the system.

## 4. Results

### 4.1. Case study: entropy-driven catalyst

**Figure 6.**
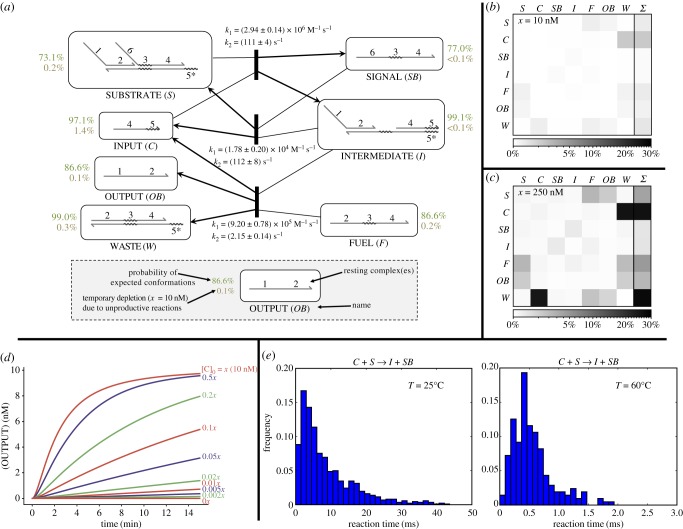
Analysis of the Zhang *et al.* entropy-driven catalyst [7] with published sequences, which are shown in figure 7*a*. All simulation data were collected at 25°C with $[{\text{Na}}^{+}]=1\;\text{M}$. Full simulation parameters are given in electronic supplementary material, appendix B. (*a*) Reaction rates and thermodynamic data for each productive reaction and predicted resting macrostate. Shown with each resting macrostate is the probability of being well formed 1 − *p*_s_ (green) and the temporary depletion bound *α*_*A*_ (brown). *p*-approximations were evaluated with *p* = 0.7, chosen because if more than 30% of the bases in the predominant long domains (16 nt) are incorrectly unbound, a spurious toehold of length 5 nt could open, which is longer than true toehold 3. (*b*) Bounds for temporary depletion of each resting macrostate (rows) due to the unproductive reaction with each other macrostate (columns) and total temporary depletion. Darker cells indicate higher depletion, which is undesired behaviour. Low depletion levels (less than 1.5%) indicate this is unlikely to affect overall system behaviour. Simulations with the unproductive reactions explicitly removed (not shown) support this prediction. Note that unequal concentrations of each resting macrostate were used, so the depletion matrix is not necessarily symmetric. Maximum concentrations were set at *c*_*C*_ = 1 nM, *c*_*F*_ = 13 nM, and all other complexes were bounded by 10 nM. These concentration limits are motivated by the concentrations in fig. 1D of [7]. (*c*) Depletion matrix with all species at 250 nM maximum concentration. Temporary depletion reaches almost 30% for some resting macrostates at this raised concentration. This motivates the use of low working concentrations for strand-displacement circuits. (*d*) Mass-action simulations of the circuit at varying initial concentrations of catalyst *C*. These demonstrate the catalytic nature of the circuit, in which the concentration of *C* determines the rate of *OB* production but not its final level. (*e*) Simulated reaction times for the first step of the catalytic cycle at 25°C and 60°C. In this case, reaction times increasingly violate an exponential distribution at higher temperatures. Note that standard mass-action chemical kinetics assumes exponentially distributed reaction times.

In this case study, we demonstrate the usage of KinDA to gain broad information about the behaviour of an entire DNA strand-displacement circuit. We perform a full analysis of the entropy-driven catalyst [7] (figure 2*d*), including every resting macrostate and both productive and unproductive reactions. Results are shown in figure 6.

Figure 6*a* shows the behaviour of each resting macrostate and each productive reaction. The rate constants for each of the three productive reactions indicate that the circuit is likely to behave as designed. The reversible first step of the catalytic cycle (*C* + *S* → *I* + *SB*) is strongly biased in the forward direction because the *k*_1_ constants differ by two orders of magnitude. The second step of the catalystic cycle (*I* + *F* → *C* + *OB* + *W*) is also biased in the forward direction, both because the *k*_1_ for the final entropy-driven reaction is higher than that of the reverse of the first step and because a high initial *F* concentration (1.3 *x*)^14^ is used. The *k*_2_ rate for this final step is the slowest of the three reactions; this is likely because the spontaneous dissociation of 6-nt toehold 5 is relatively slow.^15^ These sequences produce well-behaved resting macrostates, each with high probability (more than 70%) of adopting an enumerated domain-level conformation (for *p*-approximations with *p* = 0.7) and with low temporary depletion (less than 1.5%). While this behaviour holds at low concentrations of *x* = 10 nM (figure 6*b*), at higher concentrations of *x* = 250 nM the temporary depletion reaches almost 30% for the catalyst *C* (figure 6*c*), which would begin to affect overall kinetics. This depletion is due to *toehold occlusion* by *W* (e.g. [42]), whereby the shared toehold between these two complexes effectively sequesters *C* when bound to *W*.

Figure 6*d* shows mass-action simulations of the full system based on the KinDA-derived rate constants (see electronic supplementary material, table S2 for a full list). These simulations demonstrate the catalytic circuit behaviour observed by Zhang *et al.* [7], in which any amount of catalyst *C* produces output *OB* with rate dependent on [*C*]. Rate dependence on temperature of a single reaction is shown in figure 6*e*, which shows that at high temperatures the reaction times increasingly violate an exponential distribution. This indicates that branch migration, a non-exponentially distributed random walk process, becomes a more dominant rate-determining step at high temperatures. Note that although the qualitative circuit behaviour is correct, KinDA’s predicted reaction rates differ from those observed experimentally by roughly a factor of 4–6, as seen by circuit half-completion times (electronic supplementary material, table S3). The accuracy of the particular rates is highly dependent on Multistrand’s kinetic model, which currently does not account for important factors such as base-pair stacking at nicks. Future improvements to Multistrand [57] will produce more accurate timescale estimates by KinDA.

**Figure 7.**
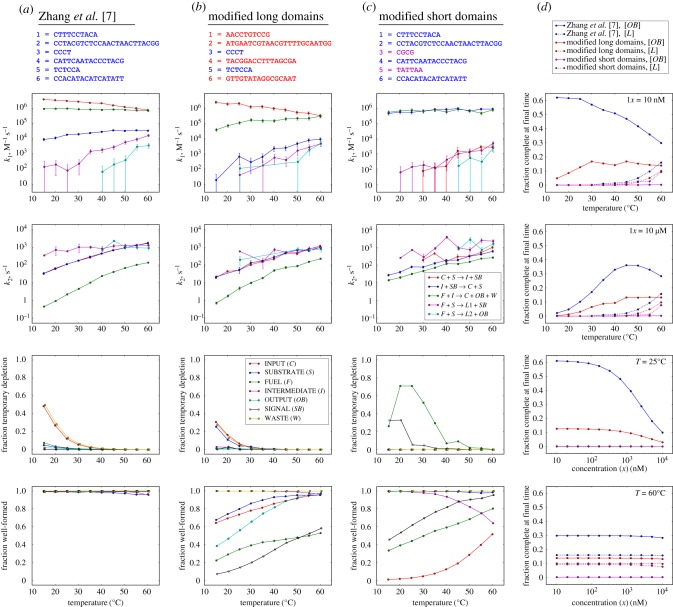
Systematic analysis of the temperature, concentration, and sequence dependence of an entropy-driven catalyst. (*a*) Original sequences from Zhang *et al.* [7]. Top plots show rate constants for the three desired reactions and two leak reactions, bottom plots show KinDA’s bound on temporary depletion for 100 nM maximum concentrations of each species and KinDA’s thermodynamic estimate of the fraction of conformations that are valid 0.51-approximations of the domain-level resting macrostates. See (*b*) and (*c*) for legends. (*b*) Sequences with modified branch migration domains, shown in red. (*c*) Sequences with modified toehold domains, shown in magenta. (*d*) Simulations of the full set of reactions according to deterministic mass-action chemical kinetics using the rate constants determined by KinDA. The CRNs considered the three desired reactions, two leak reactions and 28 unproductive reactions; reactions for which Multistrand did not encounter a successful trajectory were omitted from the CRN for the relevant case. For standard concentration *x*, the initial concentrations of species were [C]=0.1x, [F]=1.3x, [S]=1.0x. To compensate for reactions being faster at higher concentrations, the final time of a given simulation was $t_{\mathrm{final}}=15(10\;\text{nM}/x)$ min. For each sequence design, the final fractions [*OB*]/*x* and [*L*]/*x* are plotted, where [*L*] = [*L*1] + [*L*2] is the total concentration of spurious leak complexes.

Despite the limitations of the current Multistrand kinetic model, KinDA can provide important semi-quantitative insights about DNA circuit performance under conditions that were not yet experimentally investigated. Figure 7 shows an analysis of the Zhang *et al.* entropy-driven catalyst [7] at different temperatures and different concentrations, as well as comparison to systems with modified domain sequences. By performing Multistrand simulations at different temperatures, we can observe trends in system performance measures (figure 7*a*). Notably, the bimolecular rate constant *k*_1_ decreases with temperature for the reaction with the longer toehold (*C* + *S* → *I* + *SB*), has little temperature dependence for both reactions with the shorter toehold (*I* + *F* → *C* + *OB* + *W* and *I* + *SB* → *C* + *S*), but increases with temperature for the two ‘zero toehold’ leak reactions (*S* + *F* → *L*1 + *SB* and *S* + *F* → *L*2 + *OB*)^16^, where *L*1 and *L*2 are KinDA-generated strand-level complexes corresponding to these two leak pathways. By contrast, the unimolecular step’s rate constant for the same reactions, *k*_2_, increases with temperature in all cases.^17^ These trends can be understood using a phenomenological model for toehold-mediated strand displacement [58,59] in which an incoming strand, *A*, binds to a toehold of length *n* on the substrate, *B*, to form a complex, *M*, that may subsequently either complete branch migration to produce *X* and *Y* or else dissociate back into *A* and *B*:

$$A+B\overset{k_{f}}{\underset{k_{r}}{⇌}}M\overset{k_{b}}{\to }X+Y.$$

All else being equal, one would expect *k*_f_ to have little temperature dependence, *k*_b_ to scale with the speed of branch migration, which in the Multistrand model requires a single base pair to break and thus scales as $e^{\Delta G_{\mathrm{bp}}/RT}$, and *k*_r_ to scale with the rate of dissociation for a typical length *n* duplex, which in the Multistrand model requires *n* base pairs to break and thus scales as $e^{n\Delta G_{\mathrm{bp}}/RT}$, where Δ*G*_bp_ < 0 is the energy of formation for a single base pair. Phenomenologically, *k*_b_ ≈ *k*_r_ for the longer toehold at 25°C. In this model,

$$k_{1}=k_{f}\frac{k_{b}}{k_{b}+k_{r}}\;\text{and}\;k_{2}=k_{b}.$$

For longer toeholds, *k*_b_ dominates *k*_r_ at lower temperatures, but *k*_r_ has a stronger dependence on temperature than *k*_b_, speeding up dramatically at high temperatures and thus causing *k*_1_ to decrease as the fraction of successful collisions drops. By contrast, for shorter (or absent) toeholds, *k*_r_ dominates *k*_b_ at all temperatures, and thus *k*_1_ increases as *k*_r_ decreases. As for the complexes themselves, KinDA’s bound on temporary depletion was so low at the experimentally demonstrated approximately 10 nM concentrations that we performed calculations at 100 nM where temporary depletion is more significant, and even then it becomes significant only for *C* + *W* and only at low temperatures. At all temperatures, there was an insignificant fraction of poorly formed secondary structures, using the default 0.51-approximation standard. Overall, this analysis suggests that the sequences in Zhang *et al.* [7] were well designed.

The phenomenological model, used above to provide an intuitive quantitative understanding of the temperature dependence of reaction rate constants, relies on a number of assumptions that may or may not hold, depending on sequence quality. The sensitivity of the entropy-driven catalyst design to sequence choices is made clear by KinDA’s analysis of two variant systems with identical domain-level structure but modified domain sequences. Figure 7*b* considers a system where the four long domains have been replaced by random sequences using all four nucleotides, in contrast to the original sequences that (mostly) consisted of just A, T and C—a choice intended to reduce intramolecular secondary structure as well as spurious interactions between single-stranded species. Indeed, the fraction of well-formed complexes is considerably lower, even with the forgiving 0.51-approximation standard. Nevertheless, the rate constants for the three designed reactions are quantitatively similar for the two designed forward reactions, which are not much more than 10 times slower, although the designed reverse reaction (which is not essential for function) is up to 1000 times slower. Orthogonally, figure 7*c* considers a system where only the two toehold sequences have been modified, intentionally strengthening the shorter toehold while weakening the longer toehold. Not only are the complexes now poorly formed, with the catalyst *C* forming an unexpected hairpin and the fuel *F* being depleted by dimerization (as confirmed by NUPACK [45]), but now the initial reaction in the pathway (C+S→⁡I+SB) is 2–4 orders of magnitude slower than for the original sequences.

Altogether, for each design and each temperature, KinDA used Multistrand to obtain rate constants for the three intended reactions, two leak reactions and all 28 unproductive reactions. As each reaction is modelled with two elementary steps (e.g. $A+B\overset{k_{1}}{\to }C$ and $C\overset{k_{2}}{\to }D+E$), this results in a formal CRN with 66 reactions that can be simulated according to deterministic mass-action chemical kinetics to study how the various factors interact to yield an observable, such as the production of the output species. In figure 7*d*, we examine the performance of an input catalyst with an initial relative concentration of 0.1*x*, i.e. one-tenth the initial concentration of the substrate. For the experimental concentrations (*x* = 10 nM), increasing temperature slows down the original design roughly twofold, presumably largely due to C+S→⁡I+SB. The design with modified long domains, in contrast, is overall slower, but speeds up by roughly twofold. In both designs, leak accelerates at higher temperatures, approaching parity with the designed pathways by 60°C. In the design with modified toeholds, no output is produced, except through leak. An analogous set of CRN simulations, but for higher concentrations (*x* = 10 µM), reveals dramatically different phenomena: all designs have little output at low temperatures, initially increasing with temperature for the original and long-domain-modified designs. A natural hypothesis would be that the slow behaviour at low temperature is due to spurious interactions (secondary structure or temporary depletion) that are melted at higher temperatures. At first, this seems consistent with simulations that systematically increase concentrations: at 60°C, the amount of output produced is consistent with an effective bimolecular reaction, while at 25°C, less-than-expected output is produced at higher concentrations where temporary depletion must increase. Fortunately, representing the system as a CRN allows us to test this hypothesis by ‘turning off’ the 28 unproductive reactions. Simulation of this reduced CRN, which by construction has no temporary depletion, yields almost identical plots (data not shown), and points toward an alternative hypothesis: that at high concentrations reaction the pathways becomes rate-limited by the unimolecular step of $I+F\overset{k_{2}}{\to }C+OB+W$, a hypothesis that can be easily confirmed.

In summary, KinDA provides powerful tools for examining the sequence-dependence and temperature-dependence of complex strand-displacement systems. By representing the systems as CRNs, KinDA opens up the possibility of extensive system-level analysis that sheds light both on underlying biophysical principles and system-level considerations. This understanding can help identify how specific sequence-level choices can be used to optimize circuit designs.

### 4.2. Case study: multiple desired pathways

**Figure 8.**
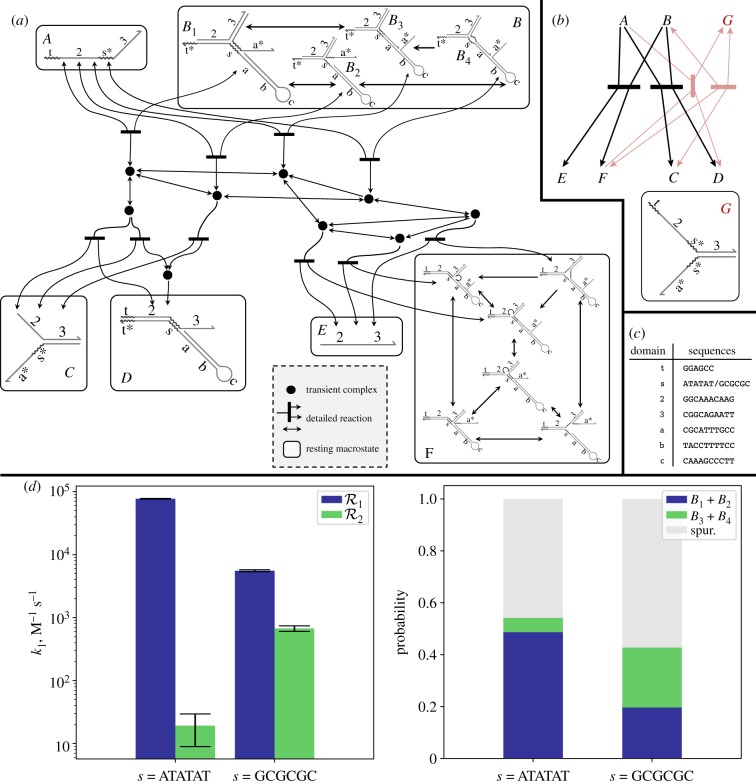
Analysis of a system with two intended condensed reactions occurring in parallel. (*a*) The detailed domain-level reaction subnetwork between complexes in resting macrostates *A* and *B*, enumerated by Peppercorn. Three product multisets are possible: {|*C*, *D*|}, {|*E*, *F*|} and {|*A*, *B*|}. The full reaction network (not shown) contains 269 complexes and 1660 reactions. (*b*) Complete condensed reaction network (top), which includes additional reactions involving an unexpected enumerated resting macrostate *G*. *G* is produced only by reactions involving the products of the desired pathways (*C*, *D* and *F*). (*c*) Sequences used for this case study. Except for domain *s*, sequences were randomly generated from a four-letter alphabet. (*d*) Bimolecular rate constant *k*_1_ for the original and modified system. Although $k_{1}^{2}$ is extremely low relative to $k_{1}^{1}$ in the original system, increasing the toehold strength significantly reduces the difference between $k_{1}^{1}$ and $k_{1}^{2}$. The conformation probabilities (right) correlate with *k*_1_ values for each pathway. This is expected behaviour because conformations *B*_1_ and *B*_2_ more easily follow $R_{1}$ while *B*_3_ and *B*_4_ more easily follow $R_{2}$. Conformation probability error bars (not shown) are insignificant.

The power of KinDA comes from its general-purpose formulation and ability to automatically analyse DNA strand-displacement circuits involving molecular complexes with arbitrary non-pseudoknotted secondary structure. Why this generality requires careful treatment of transient complexes, resting macrostates, detailed and condensed network enumeration, and interactions with multiple possible outcomes is well illustrated by the example shown in figure 8. Here, we use KinDA to analyse a system adapted from [38] in which two resting macrostates (*A* and *B*), may bind and fall into one of two *fates*, $P_{1}=\{|C,D|\}$ or $P_{2}=\{|E,F|\}$ (figure 8*a*). Figure 8*b* shows the full condensed reaction graph.^18^ Sequence-level analysis of each pathway is required to estimate which pathway, if any, is favoured. Explicitly, the two pathways we will analyse here are

$$\begin{array}{rl} & R_{1}\;:\;A+B\to C+D\\ & R_{2}\;:\;A+B\to E+F. \end{array}$$

We consider two sequence variants, one with *s* set to weaker (AT-rich) sequence *s*_w_ = ATATAT and one with *s* set to stronger (GC-rich) sequence *s*_s_ = GCGCGC. Figure 8*c*,*d* shows the sequences, their *k*_1_ rate estimates, and the conformation probabilities. While both reactions do occur with the original sequences, the rate of reaction $R_{2}$ is slower than $R_{1}$ by more than three orders of magnitude. To see why, we can analyse the resting macrostate *B* in more detail. *B* contains four resting complexes, two of which favour following $R_{1}$ and two of which favour $R_{2}$. The conformation probabilities for each resting complex are shown in figure 8*d*, right. (Here, we relax our similarity requirement to *p*-approximation with *p* = 0.51, because with a higher value of *p* many intermediates of branch migration would be classified as spurious conformations, whereas domains with partial branch migration will differ from an enumerated conformation by at most 50% and thus most will be accepted as a valid approximation when *p* = 0.51.) Using weaker toehold *s* = *s*_w_, the total probability of either *B*_1_ or *B*_2_, which favour $R_{1}$, is about 10 times greater than that of *B*_3_ or *B*_4_. The probabilities are an indicator of the bias of the system towards one pathway, although they do not account for *B* converting between its forms after *A* has bound and begun branch migration.

Using the relationship between the conformation probabilities and the relative favourabilities of $R_{1}$ and $R_{2}$, we can attempt to redesign the sequences to alter the magnitude of the system’s bias. Reasoning that one reason for the bias is the open loop at the three-way intersection of the three strands, which provides a strong entropic bias for certain conformations, we might try to counteract this effect via the strength of the toehold *s*. Figure 8*d* shows that, as expected, using the stronger toehold sequence *s* = *s*_s_ weakens the bias against $R_{2}$, although pathway $R_{1}$ is still favoured. This indicates some limits to sequence design alone; however, with reaction schemes that are highly sensitive to relative reaction rates, the ability to tune these rates quickly provides an important benefit.

### 4.3. Case study: mechanisms combining three-way and four-way branch migration

**Figure 9.**
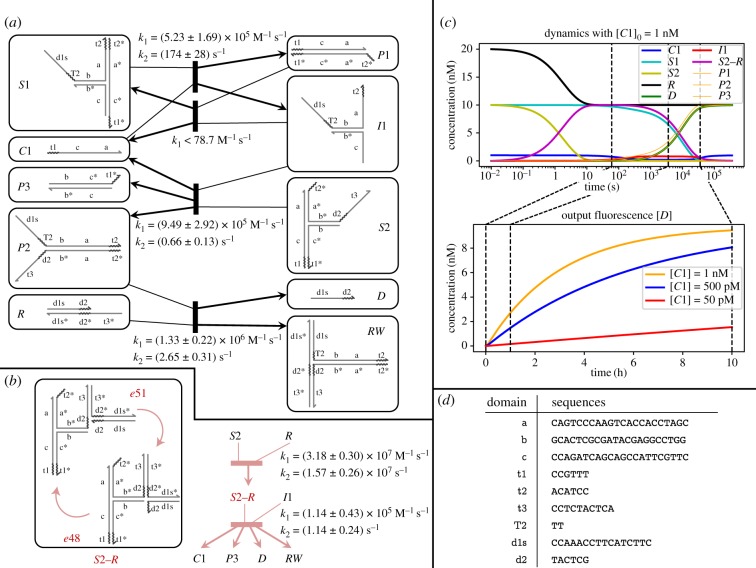
KinDA analysis of a catalytic system presented in [12], which combines three-way and four-way branch migration, simulated here at 55°C. (*a*) Domain-level complexes and reaction arrows describing the intended dynamics of the system, as well as the sequence-level reaction rates calculated by KinDA. (*b*) An unintended enumerated reaction pathway involving the resting macrostate *S*2–*R*, which contains two domain-level complexes (*e*48 and *e*51), as well as the sequence-level reaction rates calculated by KinDA. (*c*) Simulations with initial concentrations [*S*1] = [*S*2] = 20 nM, [*R*] = 30 nM. The top plot uses [*C*1] = 1 nM to illustrate the dynamics of the full system. The bottom plot compares three different initial concentrations of *C*1, for comparison with experimental data in fig. 2 of [12]. The dotted lines are at 60 s, 1 h and 10 h to help compare the different timescales used in each plot. (*d*) DNA sequences used for each domain.

KinDA can also be used to study DSD systems with complex domain-level reaction pathways that include both three-way and four-way branch migration. In figure 9, we simulate sequence-level dynamics of a catalyst system presented by Kotani & Hughes [12]. This system, shown in figure 9*a*, is more robust against leakage reactions, but includes both three-way and (generally slower) four-way branch-migration reactions. Domain-level reaction enumeration reveals that the two complexes *S*2 (the second substrate) and *R* (the reporter), both initially present at high concentrations, can interact with a 10 nt ‘toehold’, which is effectively irreversible. The result is a new resting macrostate *S*2–*R* (figure 9*b*). The subsequent depletion of the reporter complex can become a problem if there are multiple competing pathways, but as we can see in figure 9*c* the qualitative dynamics of the catalyst system in isolation is not affected. Note that *k*_1_ for the formation of *S*2–*R* is an order of magnitude faster than the fastest intended reaction, emphasizing the dominance of this reaction pathway; the *k*_2_ rate constant is even more exceptional, reflecting that this pathway just requires zippering of a helix to complete, whereas the intended reactions require some form of branch migration. On the other hand, the results show that the ‘valid’ reaction *P*1 + *I*1 → *S*1 + *C*1, which was enumerated by Peppercorn but appropriately not mentioned in [12], has an exceptionally small rate. The given *k*_1_ value is only an estimated upper bound, as out of 5 million simulated trajectories starting with complexes *P*1 + *I*1, none reached complexes *S*1 + *C*1.

The KinDA set-up for this system is as follows: we have truncated the nucleotide sequence of the reporter complex so that domain *d*1*s* is 2 nt shorter on its 5’ end than *d*1 used in [12]. This simplified the system specification, as *d*1*s* is used throughout the rest of the system. All other sequences are the same as presented in the experimental study (figure 9*d*). The simulation temperature is at 55°C (for experimental data at 25°C, see [12]). Higher temperatures make the simulation computationally feasible by speeding up reactions, notably the toehold dissociation events that often dictate the number of simulation steps before success and the probability of success itself. While it is difficult in general to infer DNA dynamics between different temperatures, the effects on branch migration with perfectly complementary sequences approximates the experimentally observed qualitative system dynamics reasonably well. The simulations use ordered-complex simulation mode for all but the ‘unintended’ reaction S2+R→S2−R, whose simulations use the stricter count-by-domain mode for reasons explained in the next section. All rates have relative errors below 40%, with the exception of the unobserved reverse reaction.

### 4.4. Case study: binding reactions and macrostates

**Figure 10.**
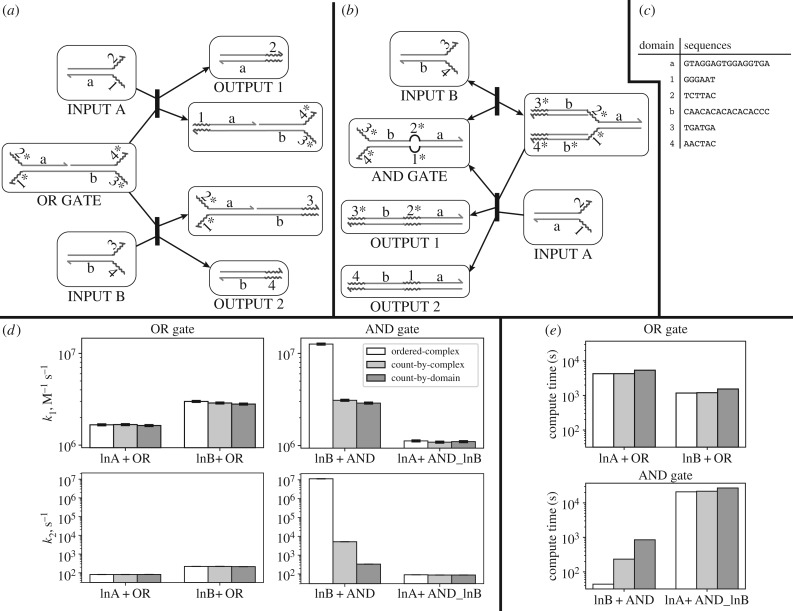
KinDA analysis of the effect of simulation stop condition modes on the Groves *et al*. [60] logic gates. (*a*) Condensed reaction network for the OR gate. (*b*) Condensed reaction network for the AND gate. (*c*) Sequences for each domain, taken from Table S3 of [60]. (*d*) Estimated reaction rates *k*_1_ and *k*_2_ for each step of each gate. Only the first step of the AND gate, which does not involve dissociation, is affected by the simulation stop mode. Simulation parameters are given in electronic supplementary material, appendix E. (*e*) Compute times for data in (*d*). Simulations were performed on a 36-core AWS machine. Note that the effect of simulation mode on compute time will also depend on the size of the DNA complexes involved.

The modes *ordered-complex*, *count-by-complex*, and *count-by-domain* (definition 3.14) modify the simulation stop conditions used by Multistrand. The count-by-domain and count-by-complex modes force the simulated complexes to more closely resemble the expected products before a trajectory halts. However, they increase compute time both because more reaction steps must be simulated and because at each step a more complex comparison is required to determine whether the simulation should halt. Using the system of logic gates designed by Groves *et al.* [60] (figure 10*a*,*b*), we can illustrate the effect of each mode on the rate estimates and compute time. This system describes two logic gates implementing OR and AND logic. In particular, the initial step of the AND gate does not involve a dissociation step; thus, simulations in ordered-complex mode will halt immediately after the two reactants initially bind. This hides the fact that, in many cases, the two complexes will immediately dissociate after binding without ever performing the subsequent four-way branch migration. By contrast, the second step of the AND gate and both steps of the OR gate involve dissociation steps and are not subject to this complication. Sequences are taken from Table S3 of [60] and shown in figure 10*c*.

Figure 10*d* shows the KinDA-derived rate estimates for *k*_1_ and *k*_2_ for each step of each gate. For the AND gate’s first reaction, the *k*_1_ rate decreases by approximately a factor of two from ordered-complex mode compared to the other modes. This decrease is likely due to the lower probability of successfully completing the reaction after binding. The *k*_2_ rate decreases dramatically as well because more reaction time steps must be simulated to reach a state satisfied by count-by-domain than by count-by-complex or ordered-complex. The corresponding increase in compute time required for this reaction (figure 10*e*) justifies the inclusion of ordered-complex mode for cases when the improved accuracy of the other modes is not required. Importantly the other reactions, which involve dissociation steps, produce very similar rate estimates in each mode. This indicates that ordered-complex mode is an appropriate approximation for reactions of this type.

## 5. Conclusion

In designing DNA strand-displacement systems, a successful system is generally highly dependent on having correct reaction rates, which ultimately determine whether molecules will take on the proper secondary structure and interact with each other in the proper ways. In a domain-level system, acceptable values of these rates are assigned to the various domain-level reactions in the system without concern for how to generate sequences that will achieve those rates. Sequence design remains a difficult problem, despite decades of work and significant advances [1,44,45,61,62], in part because satisfying thermodynamic criteria has proven to be computationally more tractable than satisfying kinetic criteria, and the thermodynamic models are more accurate than existing kinetic models, so that researchers interested in controlling the kinetics of reactions are often left using heuristics and special-case solutions [24,63,64].

The KinDA framework allows a researcher to estimate important parameters of the sequence-level system, using a general-purpose kinetics model that is continuing to improve, and determine if the sequences chosen will result in a properly functioning system. These methods make it possible to verify the kinetics resulting from a system’s sequence assignments and find the source of potential problems to determine where sequence changes are needed. Scores such as those outlined in §3.6 allow automated judgement of sequence quality.

This paper has shown how the KinDA framework may be applied to a variety of non-trivial DNA circuits. In particular, the framework was used to verify a sequence-level system’s overall behaviour by estimating kinetics for a system’s reactions and performing mass-action ODE simulations based on the first-step CRN (§§4.1 and 4.3). The framework was demonstrated in the context of complex domain-level system architectures, such as macrostates with multiple conformations (§§4.2 and 4.3); macrostate collisions with multiple potential fates (§4.2); macrostate interactions with multiple pathways towards achieving a final set of products (§§4.1–4.3); and reaction networks involving four-way and remote-toehold branch migration steps (§§4.3 and 4.4). The framework was also used to debug unexpected behaviour not predicted at the domain level: for instance, identifying productive reactions that are unacceptably slow, due to spurious conformations or temporary depletion (§§4.1 and 4.2); and discovering spurious reactions, such as leak pathways (§4.1). Finally, this paper applied the framework to evaluate the effect of different sequence choices, a basic and fundamental sequence design challenge (§4.1).

It is important to note that the accuracy of these methods is dependent on the accuracy of the underlying sequence-level simulation software used. Improvements to software like Multistrand to better match experimental data are ongoing, and recent advances to the Multistrand’s rate model (using a reduced and more tractable state space) have allowed reaction rate estimates to be improved to within a factor of 3 × for 78.5% of reactions in a comprehensive study [57]. However, this study has noted shortcomings to its model, such as failing to account for sequence-specific rigidity differences in hairpin loops and the initiation energy cost of branch migration. Future improvements to Multistrand can easily be incorporated into KinDA with few, if any, changes. Additional modifications are expected to improve Multistrand’s ability to characterize rare events efficiently, for example, by applying Markov chain methods like forward flux [65], energy barrier estimation [66] and finite state projection [67]. We are hopeful that future advances in sequence-level simulation methods will improve the efficacy of these methods.

KinDA may find utility as part of a fully automated sequence design framework that accounts for kinetics as well as thermodynamics. For instance, it could be integrated into a pipeline that uses KinDA to verify potential sequence assignments proposed by NUPACK’s thermodynamic sequence design and verification capabilities. In the long term, there is active research towards developing a nucleic acid circuit design pipeline to generate complete sequence-level system specifications by ‘compiling’ statements of high-level circuit behaviour into the machine code of nucleic acid computation. Compilers like Nuskell [42] require robust sequence verification tools such as KinDA. We hope that future work will apply our methods to continue to make automated circuit design more tractable for complex systems.

## Supplementary Material

Technical Appendix

## Endnotes

1 This class of systems includes some that, despite the name, do not make use of strand displacement.

2 With user guidance, certain unimolecular reactions may also be classified as slow, which is often necessary when reversible binding intermediates are long-lived and should be available to react with other complexes. This allows effectively trimolecular interactions, such as those underlying ‘cooperative strand displacement’ [14], to be modelled as a pathway involving bimolecular and unimolecular reactions.

3 Note that a resting macrostate is not a collection of interacting complexes but rather a collection of different conformations of a single complex that may readily convert to each other.

4 Two strands with the same sequence of domains are distinct if they have different identifiers. Thus, a modified strand (e.g. with a fluorophore) is distinguishable from an unmodified strand with the same domains.

5 At both the domain and sequence levels, we only consider non-pseudoknotted secondary structures, which have a well-nested dot-parens-plus notation representation (i.e. assuming domains are numbered sequentially then if domains *d*_*i*_ and *d*_*j*_ are bound, *d*_*k*_ and *d*_*l*_ are bound, and *i* < *k* < *j*, then *i* < *k* < *l* < *j* or *i* < *l* < *k* < *j*).

6 Strand-level complexes are equivalent to *ordered complexes*, as defined by NUPACK [45]. In that work, a *complex* referred to a molecular component involving the same set of strands but irrespective of strand order, whereas in this work we use *complex* to refer to a specific ordered complex with a specific secondary structure at the domain level or at the sequence level.

7 Two domains may only unbind spontaneously if they are *short*, as determined by the reaction enumerator. Short domains are also called toeholds.

8 The first-step model is generalized to reactions with any number of reactants, although KinDA only handles unimolecular and bimolecular reactions.

9 Although this shorthand resembles a condensed reaction, the set of condensed reactions from the enumeration may not include all unproductive reactions. In fact, Peppercorn will never include unproductive reactions when producing the condensed network. Therefore, KinDA automatically lists and considers the unproductive reactions itself.

10 Future advances to the domain-level reaction subnetwork may obviate the need for the strand-level reaction subnetwork, as would be necessary to accommodate molecules with multiple distinct resting macrostates.

11 The converse is not necessarily true, as dissociations not in strand-level reaction subnetwork may not be flagged as spurious. For instance, for a reactant *A:B:C:D* (*A*, *B*, *C*, and *D* are strands) with two enumerated strand-level reaction pathways *A:B:C:D* → *A* + *B:C:D* → *A* + *B* + *C:D* and *A:B:C:D* → *B* + *A:C:D* → *B* + *C* + *A:D*, the transition *B* + *A:C:D* → *A* + *B* + *C:D* would not render a trajectory spurious by the definition implemented in KinDA.

12 In some cases, the Boltzmann sampled initial states of *A* and *B* will have no available ways to bind with each other. Such a trajectory will halt immediately and will not be classified as any of the three reaction types in the first-step model, but will be tallied as part of the rate estimate for relevant reactions, as described in [48] and §3.5.

13 KinDA uses *k*_scale_ = 1000 by default.

14 In this section, we use *x* for a standard concentration to more easily discuss scaling concentrations of all species.

15 Although the reverse reaction of the first step (*I* + *SB* → *C* + *S*) also involves dissociation of toehold 5, successful reverse reaction trajectories are exactly those that are fast enough to prevail over the competing unproductive reaction that involves dissociation by toehold 3. Thus, the kinetics of dissociation by toehold 3 dictate the *k*_2_ for this reaction.

16 Both of these leak reactions will eventually produce *OB* + *SB* + *W*, but KinDA stops Multistrand simulations as soon as an off-pathway strand-level complex is encountered, thus distinguishing trajectories for which *SB* is release first from those for which *OB* is released first. The less probable pathway was only encountered for *T* ≥ 40°C.

17 It may seem remarkable that *k*_2_ for *C* + *S* → *I* + *SB* and for *I* + *SB* → *C* + *S* are apparently identical, despite their *k*_1_ values being different by roughly two orders of magnitude. However, it is a necessary consequence of flux balance between macrostates in an equilibrium system whose microscopic rates satisfy detailed balance, as is the case with the Multistrand’s kinetic model and FSM's Boltzmann sampling of initial conformations.

18 Note that Peppercorn has enumerated an unexpected resting macrostate not shown in figure 8*a*, *G*. This unintended domain-level feature could be analysed by KinDA as with any other enumerated feature.

## Data accessibility

This article has no additional data.
