# Supplementary material for: Automated sequence-level analysis of kinetics and thermodynamics for domain-level DNA strand-displacement systems
Source: J R Soc Interface. 2018 Dec 19;15(149):20180107. doi: 10.1098/rsif.2018.0107 (PMC6303802; doi:10.1098/rsif.2018.0107)
Supplement: Technical Appendix [file rsif20180107supp1.pdf]

Automated Sequence-Level Analysis of Kinetics and Thermodynamics for Domain-Level DNA Strand-Displacement Systems: Supplementary Information (Technical Appendix)

Joseph Berleant[‡], Chris Berlind[§], Stefan Badelt, Frits Dannenberg, Joseph Schaeffer[¶], and Erik Winfree[‖]

California Institute of Technology, Pasadena, CA, USA,
winfree@caltech.edu

A Derivations of statistical estimators

A.1 Conformation probability

The probability that, under Boltzmann equilibrium conditions, a DNA complex will when sampled adopt a sequence-level conformation corresponding to the ith domain-level conformation is estimated empirically by sampling sequence-level conformations from the Boltzmann equilibrium distribution and tabulating the number of such conformations corresponding to the domain-level conformation in question. This estimate is computed using two pieces of information: the total number of samples, N, and the number of samples corresponding to the domain-level conformation, N_i.

The Bayesian estimate for the conformation is appropriate because the maximum likelihood approach produces misleading results when $N_i = 0$ or N, which can occur when N is small compared to $\frac{1}{p_i}$ or $\frac{1}{1-p_i}$, respectively.

We use a uniform prior distribution on p_i, so $\mathrm{P}(p_i) = 1$ for all p_i. Thus, we have,

$$\hat{p}_i = \mathrm{E}[p_i|N, N_i] = \int_0^1 p_i \,\mathrm{P}(p_i|N, N_i) dp_i$$

where $\mathrm{E}[p_i|N, N_i]$ is the expectation of p_i given the observed values of N and N_i and $\mathrm{P}(p_i|N, N_i)$ is the probability mass function of p_i given the same.

We can compute $\mathrm{P}(p_i|N, N_i)$ exactly using Bayes's law and combinatorial techniques. Bayes's law states that

$$\mathrm{P}(p_i|N, N_i) = \frac{\mathrm{P}(N, N_i|p_i)\,\mathrm{P}(p_i)}{\mathrm{P}(N, N_i)}$$

where $\mathrm{P}(p_i) = 1$ and $\mathrm{P}(N, N_i) = \int_0^1 \mathrm{P}(N, N_i|p_i)dp_i$. We know that

$$\mathrm{P}(N, N_i|p_i) = \binom{N}{N_i} p_i^{N_i}(1-p_i)^{N-N_i}$$

and so

$$\mathrm{P}(N, N_i) = \binom{N}{N_i} \int_0^1 p_i^{N_i}(1-p_i)^{N-N_i} dp_i = \binom{N}{N_i} \mathrm{B}(N_i + 1, N - N_i + 1)$$

where $\mathrm{B}(\alpha, \beta) = \int_0^1 t^{\alpha-1}(1-t)^{\beta-1}dt$ is the beta function. Thus, we have

$$\mathrm{P}(p_i|N, N_i) = \frac{p_i^{N_i}(1-p_i)^{N-N_i}}{\mathrm{B}(N_i + 1, N - N_i + 1)}.$$

[‡] Current address: Massachussetts Institute of Technology, jberlean@mit.edu
[§] Current address: Oncora Medical, c.berlind@gmail.com
[¶] Current address: Autodesk Life Sciences, joseph.schaeffer@autodesk.com
[‖] To whom correspondence should be addressed, winfree@caltech.edu

$$
\begin{aligned}
\hat{p}_i =& \int_0^1 p_i \, \mathrm{P}(p_i|N, N_i) dp_i \\
=& \frac{1}{\mathrm{B}(N_i+1, N-N_i+1)} \int_0^1 p_i^{N_i+1}(1-p_i)^{N-N_i} dp_i \\
=& \frac{\mathrm{B}(N_i+2, N-N_i+1)}{\mathrm{B}(N_i+1, N-N_i+1)} \\
=& \left(\frac{(N_i+1)!(N-N_i)!}{(N+2)!}\right)\left(\frac{(N+1)!}{N_i!(N-N_i)!}\right) \\
=& \frac{N_i+1}{N+2}
\end{aligned}
$$

where we use the fact that $\mathrm{B}(\alpha, \beta) = \frac{(\alpha-1)!(\beta-1)!}{(\alpha+\beta-1)!}$ when α and β are positive integers. So we have the estimator for conformation probability p_i:

$$
\hat{p}_i = \frac{N_i+1}{N+2}.
$$

A.2 Conformation probability error

As a measure of the confidence in the estimate for p_i, we compute the standard deviation of p_i given the observed data N and N_i. We first compute the *a posteriori* variance of p_i to be:

$$
\begin{aligned}
\mathrm{Var}(p_i|N, N_i) =& \mathrm{E}[p_i^2|N, N_i] - \mathrm{E}[p_i|N, N_i]^2 \\
=& \int_0^1 p_i^2 \, \mathrm{P}(p_i|N, N_i) dp_i - \hat{p}_i^2 \\
=& \frac{1}{\mathrm{B}(N_i+1, N-N_i+1)} \int_0^1 (p_i^{N_i+2}(1-p_i)^{N-N_i} dp_i - \left(\frac{N_i+1}{N+2}\right)^2 \\
=& \frac{\mathrm{B}(N_i+3, N-N_i+1)}{\mathrm{B}(N_i+1, N-N_i+1)} - \left(\frac{N_i+1}{N+2}\right)^2 \\
=& \left(\frac{(N_i+2)!(N-N_i)!}{(N+3)!}\right)\left(\frac{(N+1)!}{N_i!(N-N_i)!}\right) - \left(\frac{N_i+1}{N+2}\right)^2 \\
=& \frac{(N_i+1)(N_i+2)}{(N+2)(N+3)} - \left(\frac{N_i+1}{N+2}\right)^2 \\
=& \frac{(N_i+1)(N-N_i+1)}{(N+2)^2(N+3)}
\end{aligned}
$$

where we again use the fact that $\mathrm{B}(\alpha, \beta) = \frac{(\alpha-1)!(\beta-1)!}{(\alpha+\beta-1)!}$ when α and β are positive integers.

The standard deviation is the square root of the variance, which is given by

$$
\hat{\sigma}_{p_i} = \sqrt{\frac{(N_i+1)(N-N_i+1)}{(N+2)^2(N+3)}} = \sqrt{\frac{\hat{p}_i(1-\hat{p}_i)}{N+3}}.
$$

A.3 k_1 estimate for bimolecular reactions

For the first-step model reaction

$$
A + B \xrightarrow{k_1^i} AB_i \xrightarrow{k_2^i} \mathcal{P}_i
$$

we estimate k_1^i using Bayesian inference on the observed simulated sequence-level reaction trajectories. Specifically, assume we observe N Multistrand trajectories, each of which begins with two conformations

of A and B sampled from the Boltzmann distribution of secondary structures. Each trajectory is characterized by an indicator variable S_i^n, which is 1 if and only if the final product multiset $\mathcal{P}_n$ equals $\mathcal{P}_i$, and its collision rate constant k_{coll}^n, which is computed as the net rate of forming any initial base pair between A and B in the first step of the simulation. For simplicity in this and the following sections, in the context of estimating k_1^i and k_2^i, we refer to trajectories in which $S_i^n = 1$ as *successful* trajectories, and all others as *unsuccessful*.

The rate constant k_1^i represents the net rate with which A and B will collide in a CME trajectory leading to $\mathcal{P}_i$, so that

$$k_1^i = \mathrm{E}\left[S_i^n\right] \mathrm{E}[k_{\text{coll},i} | S_i^n = 1] = p_i k_{\text{coll},i}$$

where $p_i = \mathrm{E}\left[S_i^n\right]$ is the probability of simulating a successful FSM trajectory and $k_{\text{coll},i}$ is the average value of k_{coll}^n over successful FSM trajectories.

The estimator $\hat{k}_1^i$ is defined as

$$\hat{k}_1^i = \mathrm{E}[k_1^i | \mathbf{P}, \mathbf{k}_{\text{coll}}] = \mathrm{E}[p_i k_{\text{coll},i} | \mathbf{P}, \mathbf{k}_{\text{coll}}]$$

where $\mathbf{P} = (\mathcal{P}_1, \mathcal{P}_2, \ldots, \mathcal{P}_N)$ and $\mathbf{k}_{\text{coll}}$ are the vectors of product multisets $\mathcal{P}_n$ and collision rates k_{coll}^n, respectively, for each of the N observed trajectories. Note that in contrast to $\mathrm{E}\left[S_i^n\right]$, which is an expectation taken over a particular distribution of trajectories, p_i and $k_{\text{coll},i}$ are underlying parameters of the system and do *not* vary between trajectories. $\mathrm{E}\left[p_i k_{\text{coll},i}\right]$ refers to an expectation taken over the space of possible $(p_i,\, k_{\text{coll},i})$, which is $[0,1] \times [0,\infty)$. This expectation is only well-defined with priors for each random variable, described below.

To make the algebra that follows more tractable, we make the simplifying assumption that p_i and $k_{\text{coll},i}$ are independent random variables, so that

$$\hat{k}_1^i = \mathrm{E}[p_i | \mathbf{P}, \mathbf{k}_{\text{coll}}]\, \mathrm{E}[k_{\text{coll},i} | \mathbf{P}, \mathbf{k}_{\text{coll}}].$$

It remains to compute the conditional expectations $\mathrm{E}[p_i | \mathbf{P}, \mathbf{k}_{\text{coll}}]$ and $\mathrm{E}[k_{\text{coll},i} | \mathbf{P}, \mathbf{k}_{\text{coll}}]$.

Observe that p_i is a random variable of the same form as a conformation probability (see Section A.1). Thus, making a similar assumption of a uniform prior on p_i over $[0,1]$, we can follow identical steps to estimate the expectation as:

$$\mathrm{E}[p_i | \mathbf{P}, \mathbf{k}_{\text{coll}}] = \frac{N_i + 1}{N + 2}$$

where N_i is the number of trajectories for which $S_i^n = 1$.

To compute the expectation on $k_{\text{coll},i}$, we first assume that the individual k_{coll}^n values for each FSM trajectory are sampled from an exponential distribution with mean $k_{\text{coll},i}$. This is justified by the fact that the exponential distribution maximizes informational entropy, so that choosing this distribution assumes the least amount of prior knowledge about the k_{coll}^n. In addition, this assumption makes the following math tractable.

By definition, we have,

$$\begin{aligned}
\mathrm{E}[k_{\text{coll},i} | \mathbf{P}, \mathbf{k}_{\text{coll}}] &= \int_0^\infty k_{\text{coll},i}\, \mathrm{P}(k_{\text{coll},i} | \mathbf{P}, \mathbf{k}_{\text{coll}}) dk_{\text{coll},i} \\
&= \int_0^\infty k_{\text{coll},i}\, \mathrm{P}(\mathbf{P}, \mathbf{k}_{\text{coll}} | k_{\text{coll},i}) \frac{\mathrm{P}(k_{\text{coll},i})}{\mathrm{P}(\mathbf{P}, \mathbf{k}_{\text{coll}})} dk_{\text{coll},i} \\
&= \frac{\int_0^\infty k_{\text{coll},i}\, \mathrm{P}(\mathbf{P}, \mathbf{k}_{\text{coll}} | k_{\text{coll},i})\, \mathrm{P}(k_{\text{coll},i}) dk_{\text{coll},i}}{\int_0^\infty \mathrm{P}(\mathbf{P}, \mathbf{k}_{\text{coll}} | k_{\text{coll},i})\, \mathrm{P}(k_{\text{coll},i}) dk_{\text{coll},i}}
\end{aligned}$$

where the second equality is due to Bayes's law and $\mathrm{P}(k_{\text{coll},i})$ is the prior probability distribution on $k_{\text{coll},i}$.

To compute the value of $\mathrm{P}(\mathbf{P}, \mathbf{k}_{\text{coll}} | k_{\text{coll},i})$, first observe that all trajectories are independent, so that

$$\mathrm{P}(\mathbf{P}, \mathbf{k}_{\text{coll}} | k_{\text{coll},i}) = \prod_{1 \leq n \leq N} \mathrm{P}(k_{\text{coll}}^n | k_{\text{coll},i}).$$

Because $k_{\text{coll},i}$ gives no information about unsuccessful trajectories, any terms due to these trajectories will be canceled out by the normalization term, and we may drop these terms. For successful trajectories,

our assumption regarding the distribution of k^n_{coll} (above) gives us:

$$\mathrm{P}(k^n_{\text{coll}}|k_{\text{coll},i}) = \frac{1}{k_{\text{coll},i}} \exp\left(-\frac{k^n_{\text{coll}}}{k_{\text{coll},i}}\right)$$

so that

$$\begin{aligned}
\mathrm{P}(\mathbf{P}, \mathbf{k}_{\text{coll}}|k_{\text{coll},i}) &= \prod_{S^n_i=1} \frac{1}{k_{\text{coll},i}} \exp\left(-\frac{k^n_{\text{coll}}}{k_{\text{coll},i}}\right) \\
&= \left(\frac{1}{k_{\text{coll},i}}\right)^{N_i} \exp\left(-\frac{\sum_{S^n_i=1} k^n_{\text{coll}}}{k_{\text{coll},i}}\right) \\
&= \left(\frac{1}{k_{\text{coll},i}}\right)^{N_i} \exp\left(-\frac{\gamma_i}{k_{\text{coll},i}}\right), \text{ where } \gamma_i = \sum_{S^n_i=1} k^n_{\text{coll}}.
\end{aligned}$$

Substituting into the equation for the expectation of $k_{\text{coll},i}$ yields

$$\mathrm{E}[k_{\text{coll},i}|\mathbf{P}, \mathbf{k}_{\text{coll}}] = \frac{\int_0^\infty \left(\frac{1}{k_{\text{coll},i}}\right)^{N_i-1} \exp\left(-\frac{\gamma_i}{k_{\text{coll},i}}\right) \mathrm{P}(k_{\text{coll},i}) dk_{\text{coll},i}}{\int_0^\infty \left(\frac{1}{k_{\text{coll},i}}\right)^{N_i} \exp\left(-\frac{\gamma_i}{k_{\text{coll},i}}\right) \mathrm{P}(k_{\text{coll},i}) dk_{\text{coll},i}}$$

To allow this expression to be evaluated analytically for all nonnegative N_i, we use the prior distribution

$$\mathrm{P}(k_{\text{coll},i}) = \left(\frac{1}{k_{\text{coll},i}}\right)^3.$$

Although this is an improper prior over $[0, \infty)$, the form of the integrand allows both the numerator and denominator of $\mathrm{E}[k_{\text{coll},i}|\mathbf{P}, \mathbf{k}_{\text{coll}}]$ to converge. First, considering the numerator,

$$\begin{aligned}
\int_0^\infty \left(\frac{1}{k_{\text{coll},i}}\right)^{N_i-1} \exp\left(-\frac{\gamma_i}{k_{\text{coll},i}}\right) \mathrm{P}(k_{\text{coll},i}) dk_{\text{coll},i} &= \int_0^\infty \left(\frac{1}{k_{\text{coll},i}}\right)^{N_i+2} \exp\left(-\frac{\gamma_i}{k_{\text{coll},i}}\right) dk_{\text{coll},i} \\
&= \int_0^\infty \frac{u^{N_i}}{\gamma_i^{N_i+1}} e^{-u} du, \text{ where } u = \frac{\gamma_i}{k_{\text{coll},i}} \\
&= \frac{N_i!}{\gamma_i^{N_i+1}}
\end{aligned}$$

where in the last equality we use the fact that $m! = \int_0^\infty x^m e^{-x} dx$ for nonnegative integral m.

Similarly, the denominator can be simplified to

$$\int_0^\infty \left(\frac{1}{k_{\text{coll},i}}\right)^{N_i+3} \exp\left(-\frac{\gamma_i}{k_{\text{coll},i}}\right) dk_{\text{coll},i} = \frac{(N_i+1)!}{\gamma_i^{N_i+2}}$$

and we have

$$\mathrm{E}[k^i_1|\mathbf{P}, \mathbf{k}_{\text{coll}}] = \left(\frac{\gamma_i}{N_i+1}\right)\left(\frac{N_i+1}{N+2}\right) = \frac{\gamma_i}{N+2}$$

A.4 k_1 error estimate for bimolecular reactions

As with conformation probabilities, the spread in k_1 given the observed trajectories can be computed as the standard deviation of the posterior distribution of k_1. That is,

$$
\begin{aligned}
\mathrm{Var}(k_1^i|\mathbf{P},\mathbf{k}_{\mathrm{coll}}) =& \mathrm{E}[(k_1^i)^2|\mathbf{P},\mathbf{k}_{\mathrm{coll}}] - \mathrm{E}[k_1^i|\mathbf{P},\mathbf{k}_{\mathrm{coll}}]^2 \\
=& \mathrm{E}[(k_{\mathrm{coll},i})^2|\mathbf{P},\mathbf{k}_{\mathrm{coll}}]\,\mathrm{E}[p_i^2|\mathbf{P},\mathbf{k}_{\mathrm{coll}}] - \left(\frac{\gamma_i}{N+2}\right)^2 \\
=& \left(\frac{\int_0^\infty (k_{\mathrm{coll},i})^2\,\mathrm{P}(\mathbf{P},\mathbf{k}_{\mathrm{coll}}|k_{\mathrm{coll},i})\,\mathrm{P}(k_{\mathrm{coll},i})dk_{\mathrm{coll},i}}{\int_0^\infty \mathrm{P}(\mathbf{P},\mathbf{k}_{\mathrm{coll}}|k_{\mathrm{coll},i})\,\mathrm{P}(k_{\mathrm{coll},i})dk_{\mathrm{coll},i}}\right)\left(\frac{(N_i+1)(N_i+2)}{(N+2)(N+3)}\right) - \left(\frac{\gamma_i}{N+2}\right)^2 \\
=& \left(\frac{\frac{(N_i-1)!}{\gamma_i^{N_i}}}{\frac{(N_i+1)!}{\gamma_i^{N_i+2}}}\right)\left(\frac{(N_i+1)(N_i+2)}{(N+2)(N+3)}\right) - \left(\frac{\gamma_i}{N+2}\right)^2 \\
=& \frac{\gamma_i^2}{N_i(N_i+1)}\frac{(N_i+1)(N_i+2)}{(N+2)(N+3)} - \left(\frac{\gamma_i}{N+2}\right)^2 \\
=& \frac{\gamma_i^2(2N-N_i+1)}{N_i(N+2)^2(N+3)}
\end{aligned}
$$

using again the fact that $\int_0^\infty x^n e^{-x}dx = n!$ for integral nonnegative n.

The width of the posterior distribution is measured by the standard deviation, or square root of the variance. So we estimate the error of the estimate for k_1 to be:

$$
\hat{\sigma}_{k_1^i} = \sqrt{\frac{\gamma_i^2(2N-N_i+1)}{N_i(N+2)^2(N+3)}} = \hat{k}_1^i\sqrt{\frac{2N-N_i+1}{N_i(N+3)}}
$$

.

Note that in the edge case with $N_i = 0$ and $N > 0$ we estimate an upper bound on k_1 as:

$$
k_1^i \leq \max_n k_{\mathrm{coll}}^n \left(\frac{N_i+1}{N+2}\right)
$$

and report error bounds as infinite.

A.5 k_2 error estimate for bimolecular reactions

Using Equation (6) of the main text, it is apparent that the unimolecular reaction rate k_2^i is computed as the weighted average of the reaction times, where weights are derived as the k_{coll}^n corresponding to each simulated reaction time τ_2^n. We first derive the variance of the mean reaction time $\tau_{2,i}$ for the ith first-step model reaction, from which the variance of k_2^i may be computed.

As previously noted, we estimate $\tau_{2,i}$ as the weighted average:

$$
\hat{\tau}_{2,i} = \frac{\sum\limits_{S_i^n=1} k_{\mathrm{coll}}^n \tau_2^n}{\sum\limits_{S_i^n=1} k_{\mathrm{coll}}^n} = \sum_{S_i^n=1} w_i^n \tau_2^n
$$

where w_i^n is the normalized weight $\frac{k_{\mathrm{coll}}^n}{\sum_{S_i^n} k_{\mathrm{coll}}^n}$ for each simulated trajectory.

We assume that all reaction times are drawn from a distribution with mean $\mu_{\tau_2^n}$ and variance $\sigma_{\tau_2^n}^2$. The variance of the *individual* τ_2^n differs from the variance of their weighted sum, $\sigma_{\tau_{2,i}}^2$. Assuming fixed weights w_i^n, this is given by

$$
\sigma_{\tau_{2,i}}^2 = \sum_{S_i^n=1} (w_i^n)^2 \sigma_{\tau_2^n}^2 .
$$

Thus, we can estimate the variance of the weighted sum from the variance from which the individual reaction times are drawn. Note that since w_i^n is actually a random variable, our estimates are neglecting this source of variability.

To estimate $\sigma^2_{\tau_2^n}$, the variance of the distribution for individual reaction times, we first propose the estimator:

$$\hat{\sigma}^2_{\tau_2^n} = \sum_{S_i^n=1} w_i^n (\tau_2^n - \hat{\tau}_{2,i})^2.$$

This estimator is biased, and the bias can be quantified by computing the expectation of $\hat{\sigma}^2_{\tau_2^n}$, again treating w_i^n as fixed:

$$\mathrm{E}\left[\hat{\sigma}^2_{\tau_2^n}\right] = \sum_n w_i^n \, \mathrm{E}\left[(\tau_2^n - \hat{\tau}_{2,i})^2\right]$$

where the summation is over only successful trajectories (i.e. for which $S_i^n = 1$). All summations in the remainder of this derivation are similarly performed over only successful trajectories.

Expanding the definition of $\hat{\tau}_{2,i}$ yields

$$\begin{aligned}
\mathrm{E}\left[(\tau_2^n - \hat{\tau}_{2,i})^2\right] =& \mathrm{E}\left[(\tau_2^n - \sum_m w_i^m \tau_2^m)^2\right] \\
=& \mathrm{E}\left[\left(\left(\sum_m w_i^m\right)\tau_2^n - \sum_m w_i^m \tau_2^m\right)^2\right] \\
=& \mathrm{E}\left[\left(\sum_m w_i^m (\tau_2^n - \tau_2^m)\right)^2\right] \\
=& \mathrm{E}\left[\sum_{l,m} w_i^l w_i^m (\tau_2^n - \tau_2^l)(\tau_2^n - \tau_2^m)\right] \\
=& \mathrm{E}\left[\sum_{l,m} w_i^l w_i^m (\tau_2^n)^2 - 2\sum_{l,m} w_i^l w_i^m \tau_2^m \tau_2^n + \sum_{l,m} w_i^l w_i^m \tau_2^l \tau_2^m\right] \\
=& \mathrm{E}\left[(\tau_2^n)^2 \sum_{l,m} w_i^l w_i^m\right] - 2\,\mathrm{E}\left[\tau_2^n \sum_{l,m} w_i^l w_i^m \tau_2^m\right] + \mathrm{E}\left[\sum_{l,m} w_i^l w_i^m \tau_2^l \tau_2^m\right] \\
=& \mathrm{E}\left[(\tau_2^n)^2\right]\left(\sum_m w_i^m\right)\left(\sum_m w_i^m\right) - 2\left(\sum_l w_i^l\right)\sum_m w_i^m \,\mathrm{E}\left[\tau_2^n \tau_2^m\right] + \sum_{l,m} w_i^l w_i^m \,\mathrm{E}\left[\tau_2^l \tau_2^m\right] \\
=& \mathrm{E}\left[(\tau_2^n)^2\right] - 2\left(\sum_{m \neq n} w_i^m \mu^2_{\tau_2^n} + w_i^n \,\mathrm{E}\left[(\tau_2^n)^2\right]\right) + \left(\sum_{l \neq n}\sum_{m \neq n} w_i^l w_i^m \mu^2_{\tau_2^n} + \sum_m (w_i^m)^2 \,\mathrm{E}\left[(\tau_2^m)^2\right]\right) \\
=& \mathrm{E}\left[(\tau_2^n)^2\right] - 2\left(\sum_m w_i^m \mu^2_{\tau_2^n} + w_i^n \sigma^2_{\tau_2^n}\right) + \left(\sum_l \sum_m w_i^l w_i^m \mu^2_{\tau_2^n} + \sum_m (w_i^m)^2 \sigma^2_{\tau_2^n}\right) \\
=& \mathrm{E}\left[(\tau_2^n)^2\right] - 2\left(\mu^2_{\tau_2^n} + w_i^n \sigma^2_{\tau_2^n}\right) + \left(\mu^2_{\tau_2^n} + \sum_m (w_i^m)^2 \sigma^2_{\tau_2^n}\right) \\
=& \mathrm{E}\left[(\tau_2^n)^2\right] - \mu^2_{\tau_2^n} - 2w_i^n \sigma^2_{\tau_2^n} + \sum_m (w_i^m)^2 \sigma^2_{\tau_2^n} \\
=& \sigma^2_{\tau_2^n} - 2w_i^n \sigma^2_{\tau_2^n} + \sum_m (w_i^m)^2 \sigma^2_{\tau_2^n} \\
=& \sigma^2_{\tau_2^n}(1 - 2w_i^n + \sum_m (w_i^m)^2).
\end{aligned}$$

Substituting into the expression for $\mathrm{E}\left[\hat{\sigma}^2_{\tau_2^n}\right]$,

$$\mathrm{E}\left[\hat{\sigma}^2_{\tau_2^n}\right] = \sum_n w_i^n \sigma^2_{\tau_2^n}(1 - 2w_i^n + \sum_m (w_i^m)^2)$$

and, simplifying,

$$\mathrm{E}\left[\hat{\sigma}^2_{\tau_2^n}\right] = \sigma^2_{\tau_2^n}(1 - \sum_n (w_i^n)^2).$$

Thus, an unbiased estimator for $\sigma^2_{\tau_2^n}$ is

$$\frac{\hat{\sigma}^2_{\tau_2^n}}{1 - \sum_n (w_i^n)^2}$$

and an unbiased estimator for $\sigma^2_{\tau_{2,i}}$ is

$$\hat{\sigma}^2_{\tau_{2,i}} = \frac{\sum_n (w_i^n)^2}{1 - \sum_n (w_i^n)^2} \hat{\sigma}^2_{\tau_2^n}.$$

So we can estimate the standard deviation of $\tau_{2,i}$ with

$$\hat{\sigma}_{\tau_{2,i}} = \sqrt{\frac{\sum_n (w_i^n)^2}{1 - \sum_n (w_i^n)^2}} \hat{\sigma}_{\tau_2^n}$$

and, using the fact that $k_2^i = \frac{1}{\tau_{2,i}}$, we make a linear approximation for the relationship between k_2^i and $\tau_{2,i}$, and propagate that to the variance:

$$\hat{\sigma}_{k_2^i} = \left(\hat{k}_2^i\right)^2 \hat{\sigma}_{\tau_{2,i}}.$$

We can further simplify this expression, by letting

$$N_{i,\mathrm{eff}} = \frac{1}{\sum_n (w_i^n)^2} = \frac{\left(\sum_n k^n_{\mathrm{coll}}\right)^2}{\sum_n (k^n_{\mathrm{coll}})^2}$$

so that

$$\hat{\sigma}_{k_2^i} = \left(\hat{k}_2^i\right)^2 \sqrt{\frac{1}{N_{i,\mathrm{eff}} - 1}} \hat{\sigma}_{\tau_2^n}$$

where

$$\hat{\sigma}_{\tau_2^n} = \sqrt{\frac{\sum_n k^n_{\mathrm{coll}} \left(\tau_2^n - \tau_{2,i}\right)^2}{\sum_n k^n_{\mathrm{coll}}}}.$$

This form of the expression shows the inverse square-root relationship with respect to the number of samples, which is characteristic of standard errors of the mean over multiple independent samples.

Unfortunately, a few of our reported plots and calculations used a previous, incorrect, version of this estimate for $\hat{\sigma}_{k_2^i}$, but the deviations were less than 5% in cases that we tested.

Finally, we note that this calculation does not account for the additional uncertainty due to variation in k^n_{coll}. Future work may aim to develop a more rigorous estimate that accounts for this source of variation.

B Case Study: Entropy-driven Catalyst [?]

The full system is given in Figure 6A (main text). In addition to the productive reactions shown there, unproductive reactions were included between every pair of resting macrostates for a total of 31 non-spurious reactions (3 productive, 28 unproductive).

Sequences are taken from [?], Table 1.

Domain	Length (nt)	Sequence
1	10	CTTTCCTACA
2	24	CCTACGTCTCCAACTAACTTACGG
3	4	CCCT
4	16	CATTCAATACCCTACG
5	6	TCTCCA
6	16	CCACATACATCATATT

Table S1. Sequences for entropy-driven catalyst. Taken from Table 1 of [?].

All Multistrand simulations were performed at 25°C with sodium concentration of 1 M, magnesium concentration of 0 M, GT wobble pairing enabled, dangles energy parameter 'Some', and Metropolis rate method. The values of k_{bi} and k_{uni} were 8.01171383×10^5 $\text{M}^{-1}\text{s}^{-1}$ and 2.41686715×10^6 s^{-1}, respectively. These values were determined as the mode of the posterior distribution after training a simplified Multistrand-like model on an extensive experimental dataset [?].

A single spurious reaction was observed between Substrate and Fuel under these parameters. We expect this spurious reaction to occur via 0-toehold strand displacement. This spurious reaction becomes more prominent at higher temperatures.

Reactants			Products				Trajectories	k_1 ($\text{M}^{-1}\text{s}^{-1}$)	k_2 (s^{-1})
Substrate	+	Catalyst	Intermediate	+	Signal		869/20881	$(2.94\pm0.14)\times10^6$	111 ± 4
Intermediate	+	Signal	Substrate	+	Catalyst		151/630000	$(1.78\pm0.20)\times10^4$	112 ± 8
Fuel	+	Intermediate	Output	+	Catalyst	+ Waste	278/20258	$(9.20\pm0.78)\times10^5$	2.15 ± 0.14
Catalyst	+	Waste	Catalyst	+	Waste		19992/20000	$(2.83\pm0.02)\times10^7$	20.5 ± 0.6
Substrate	+	Signal	Substrate	+	Signal		19997/20000	$(1.14\pm0.01)\times10^8$	$(7.33\pm0.24)\times10^4$
Fuel	+	Waste	Fuel	+	Waste		19982/20000	$(3.84\pm0.03)\times10^7$	433 ± 53
Catalyst	+	Signal	Catalyst	+	Signal		19992/20000	$(5.69\pm0.04)\times10^7$	$(7.24\pm0.70)\times10^4$
Catalyst	+	Catalyst	Catalyst	+	Catalyst		20000/20000	$(3.97\pm0.03)\times10^7$	$(1.14\pm0.10)\times10^4$
Fuel	+	Catalyst	Fuel	+	Catalyst		20000/20000	$(5.31\pm0.04)\times10^7$	$(3.34\pm0.41)\times10^4$
Fuel	+	Fuel	Fuel	+	Fuel		19999/20000	$(7.41\pm0.05)\times10^7$	$(6.50\pm0.77)\times10^4$
Waste	+	Waste	Waste	+	Waste		19977/20000	$(4.34\pm0.03)\times10^6$	$(3.39\pm0.16)\times10^6$
Substrate	+	Substrate	Substrate	+	Substrate		20000/20000	$(1.40\pm0.01)\times10^8$	$(6.87\pm0.26)\times10^4$
Signal	+	Waste	Signal	+	Waste		19989/20000	$(4.23\pm0.03)\times10^7$	$(2.81\pm0.05)\times10^4$
Output	+	Signal	Output	+	Signal		19999/20000	$(5.86\pm0.04)\times10^7$	$(4.06\pm0.70)\times10^5$
Catalyst	+	Intermediate	Catalyst	+	Intermediate		19999/20000	$(4.86\pm0.03)\times10^7$	$(9.54\pm0.55)\times10^4$
Fuel	+	Signal	Fuel	+	Signal		19998/20000	$(7.76\pm0.05)\times10^7$	$(1.93\pm0.20)\times10^5$
Intermediate	+	Signal	Intermediate	+	Signal		629803/630000	$(7.20\pm0.01)\times10^7$	$(8.82\pm0.14)\times10^3$
Signal	+	Signal	Signal	+	Signal		19994/20000	$(8.08\pm0.06)\times10^7$	$(4.22\pm0.26)\times10^5$
Substrate	+	Intermediate	Substrate	+	Intermediate		20000/20000	$(8.20\pm0.06)\times10^7$	$(9.98\pm0.50)\times10^4$
Substrate	+	Fuel	Substrate	+	Fuel		1819978/1820000	$(1.05\pm0.00)\times10^8$	$(1.02\pm0.02)\times10^3$
Output	+	Substrate	Output	+	Substrate		20000/20000	$(7.61\pm0.05)\times10^7$	$(1.45\pm0.25)\times10^3$
Intermediate	+	Intermediate	Intermediate	+	Intermediate		20000/20000	$(4.68\pm0.03)\times10^7$	$(3.13\pm0.10)\times10^5$
Output	+	Catalyst	Output	+	Catalyst		19998/20000	$(3.99\pm0.03)\times10^7$	$(5.51\pm1.00)\times10^4$
Output	+	Waste	Output	+	Waste		19988/20000	$(2.73\pm0.02)\times10^7$	597 ± 62
Output	+	Output	Output	+	Output		19996/20000	$(3.81\pm0.03)\times10^7$	$(2.03\pm0.73)\times10^5$
Substrate	+	Catalyst	Substrate	+	Catalyst		20012/20881	$(7.57\pm0.05)\times10^7$	$(8.26\pm1.31)\times10^4$
Fuel	+	Intermediate	Fuel	+	Intermediate		19980/20258	$(6.47\pm0.05)\times10^7$	$(1.86\pm0.25)\times10^4$
Output	+	Fuel	Output	+	Fuel		19995/20000	$(5.42\pm0.04)\times10^7$	$(2.15\pm0.56)\times10^5$
Substrate	+	Waste	Substrate	+	Waste		19983/20000	$(4.05\pm0.03)\times10^7$	$(4.07\pm0.13)\times10^4$
Output	+	Intermediate	Output	+	Intermediate		19997/20000	$(4.61\pm0.03)\times10^7$	$(2.47\pm0.07)\times10^5$
Intermediate	+	Waste	Intermediate	+	Waste		19985/20000	$(2.13\pm0.02)\times10^7$	$(3.27\pm0.09)\times10^4$
Substrate	+	Fuel	rs_F:LB:OB	+	rs_SB		1/1820000	78.8 ± 111.4	$603\pm\text{inf}$

Table S2. Reaction rate estimates for all productive, unproductive, and (observed) spurious reactions in the entropy-driven catalyst. The Trajectories column shows the number of trajectories corresponding to each reaction over the total number of trajectories simulated for the reactants.

$[C]_0$ (nM)	KinDA (min)	Zhang et al. [?] (min)	Ratio (Zhang et al./KinDA)
10	2.2	8.0	3.6
5	3.4	14.5	4.3
2	7.2	36.2	5.0
1	13.6	73.1	5.4
0.5	26.4	147.0	5.6
0.2	65.0	367.8	5.7
0.1	129.4	731.5	5.7
0.05	258.9	1443.0	5.6
0.02	652.7	3459.4	5.3

Table S3. System half-completion times for varying initial catalyst C concentrations. Half-completion times estimated by simulating the mass-action ODEs for the rates in Table S2 and finding the time at which $[OB]$=5 nM. Initial concentrations of S and F are 10 nM and 13 nM for all simulations. Half-completion times were also estimated using the published reaction rates in Zhang et al. [?]. KinDA overestimates the reaction rates so all half times are much lower than those calculated with the published rates, by a factor of about 4-6.

Resting Macrostate	Complex	Samples	p (%)
Input	Input	483792/500500	96.7±0.0
	spurious	16708/500500	3.34±0.03
Output	Output	431370/500500	86.2±0.0
	spurious	69130/500500	13.8±0.0
Waste	Waste	500020/500500	99.9±0.0
	spurious	480/500500	$(9.61\pm0.44)\times10^{-2}$
Signal	Signal	382146/500500	76.4±0.1
	spurious	118354/500500	23.6±0.1
Intermediate	Intermediate	496772/500500	99.3±0.0
	spurious	3728/500500	$(7.45\pm0.12)\times10^{-1}$
Substrate	Substrate	364393/500500	72.8±0.1
	spurious	136107/500500	27.2±0.1
Fuel	Fuel	433740/500500	86.7±0.0
	spurious	66760/500500	13.3±0.0

Table S4. Probabilities of p-approximations for each resting complex, with $p = 0.7$. The Samples column shows number of sampled conformations that p-approximate the resting complex (or are p-spurious) over the total number of sampled conformations.

C Case Study: Multiple Desired Pathways

The full system is given in Figure 8 (main text). The sequences were randomly generated from a four-letter alphabet with an equal probability of each base, with the exception of toeholds s_w and s_s, which were chosen to be weak and strong toeholds, respectively.

Domain	Length (nt)	Sequence
t	6	GGAGCC
$\mathrm{s} = \mathrm{s_w}$	6	ATATAT
$\mathrm{s} = \mathrm{s_s}$	6	GCGCGC
2	10	GGCAAACAAG
3	10	CGGCAGAATT
a	10	CGCATTTGCC
b	10	TACCTTTTCC
c	10	CAAAGCCCTT

Table S5. Sequences for the multiple fates case study.

For both the unmodified (with $s = s_w$) and modified (with $s = s_s$) systems, the reactions $A + B \rightarrow C + D$ and $A + B \rightarrow E + F$ were analyzed. Multistrand simulations were run with the same parameters as the entropy-driven catalyst (Appendix B).

Reactants	Products			Trajectories	k_1 ($\mathrm{M^{-1}s^{-1}}$)	k_2 ($\mathrm{s^{-1}}$)
Gate + Interloper	Fate1_Cpx1	+	Fate1_Cpx2	13532/5000000	$(7.71 \pm 0.09) \times 10^4$	$(1.54 \pm 0.02) \times 10^3$
Gate + Interloper	Fate2_Cpx1	+	Fate2_Cpx2	7/5000000	19.2 ± 10.3	862 ± 107
Gate + Interloper	Gate	+	Interloper	4976331/5000000	$(3.27 \pm 0.00) \times 10^7$	$(6.04 \pm 0.02) \times 10^5$
Gate + Interloper	rs_strand23:strandGATE	+	rs_strand123:strand3A	43/5000000	665 ± 143	$(1.66 \pm 0.40) \times 10^3$

Table S6. Reaction rate estimates for reactions in the multiple fates case study (unmodified system, $s = s_w$). The Trajectories column shows the number of trajectories corresponding to each reaction over the total number of trajectories simulated for the reactants.

Reactants	Products			Trajectories	k_1 ($\mathrm{M^{-1}s^{-1}}$)	k_2 ($\mathrm{s^{-1}}$)
Gate + Interloper	Fate1_Cpx2	+	Fate1_Cpx1	1611/5042000	$(5.55 \pm 0.20) \times 10^3$	270 ± 6
Gate + Interloper	Fate2_Cpx2	+	Fate2_Cpx1	200/5042000	672 ± 67	268 ± 18
Gate + Interloper	Gate	+	Interloper	5001511/5042000	$(1.95 \pm 0.00) \times 10^7$	$(3.93 \pm 0.02) \times 10^5$
Gate + Interloper	rs_strand23:strandGATE	+	rs_strand123:strand3A	1/5042000	3.97 ± 5.62	$491 \pm \mathrm{inf}$

Table S7. Reaction rate estimates for reactions in the multiple fates case study (modified system, $s = s_s$). The Trajectories column shows the number of trajectories corresponding to each reaction over the total number of trajectories simulated for the reactants.

Resting Macrostate	Complex	Samples	p (%)
B (unmodified)	B_1	14477/201612	7.18±0.06
	B_2	83652/201612	41.5±0.1
	B_3	11072/201612	5.49±0.05
	B_4	50/201612	$(2.53\pm0.35)\times10^{-2}$
	spurious	92361/201612	45.8±0.1
B (modified)	B_1	39381/200465	19.6±0.1
	B_2	0/200465	$(4.99\pm4.99)\times10^{-4}$
	B_3	46345/200465	23.1±0.1
	B_4	0/200465	$(4.99\pm4.99)\times10^{-4}$
	spurious	114739/200465	57.2±0.1

Table S8. Resting macrostate conformation probabilities for resting macrostate B (see main text) for both $s = s_w$ and $s = s_s$. The Samples column shows number of sampled conformations that p-approximate the resting complex (or are p-spurious) over the total number of sampled conformations.

D Case Study: Mechanisms Combining 3-way and 4-way Branch Migration

Domain	Length (nt)	Sequence
a	22	CAGTCCCAAGTCACCACCTAGC
b	22	GCACTCGCGATACGAGGCCTGG
c	22	CCAGATCAGCAGCCATTCGTTC
t1	6	CCGTTT
t2	6	ACATCC
t3	10	CCTCTACTCA
T2	2	TT
d1s	16	CCAAACCTTCATCTTC
d2	6	TACTCG

Table S9. Sequences for case study, taken from Kotani & Hughes [?]. Note that our simulations used a modified reporter complex from [?] that uses domain $d1s$ rather than $d1$. $d1s$ is produced by removing 2 nt from the 5' end of $d1$.

Reactants	Products	Trajectories	k_1 ($M^{-1}s^{-1}$)	k_2 (s^{-1})
S1 + C1	P1 + I1	19/5000	(5.23±1.70) $\times 10^5$	174±28
P1 + I1	S1 + C1	0/5000000	< 78.7	—
I1 + S2	C1 + P3 + P2	21/5000	(9.49±2.92) $\times 10^5$	0.66±0.13
P2 + R	D + RW	72/5000	(1.34±0.22) $\times 10^6$	2.66±0.32
S2 + R	S2-R	154/247	(3.18±0.30) $\times 10^7$	1.57±0.13 $\times 10^7$
I1 + S2-R	C1 + P3 + D + RW	14/25000	(1.14±0.43) $\times 10^5$	1.14±0.24

Table S10. Reaction rate estimates for the Kotani & Hughes (2017) [?] case study. All simulations were run in ordered-complex mode, except for the unexpected reaction, $S2 + R \to S2\text{-}R$, which has only one product and was run in count-by-domain mode. The Trajectories column shows the number of trajectories corresponding to each reaction over the total number of trajectories simulated for the reactants. Simulation parameters were the same as for the entropy-driven catalyst (Appendix B).

E Case Study: Binding Reactions and Flavors of Macrostates

DNA sequences were taken from Groves et al. [?]. All Multistrand simulations used the same parameters as with the entropy-driven catalyst (Appendix B).

Domain	Length (nt)	Sequence
a	16	GTAGGAGTGGAGGTGA
1	6	GGGAAT
2	6	TCTTAC
b	16	CAACACACACACACCC
3	6	TGATGA
4	6	AACTAC

Table S11. Sequences for case study, taken from Groves et al. [?].

Reactants	Products	Trajectories	k_1 ($M^{-1}s^{-1}$)	k_2 (s^{-1})
InA + OR	OR_InA_Waste + OR_InA_Sig	3160/104931	(1.66±0.04) $\times 10^6$	50.7±0.9
InB + OR	OR_InB_Waste + OR_InB_Sig	3136/59496	(2.99±0.07) $\times 10^6$	189±3
InA + OR	InA + OR	101490/104931	(4.71±0.02) $\times 10^7$	(4.85±0.23) $\times 10^3$
InB + OR	InB + OR	56360/59496	(5.01±0.02) $\times 10^7$	(4.39±0.35) $\times 10^4$

Table S12. Reaction rate estimates for the OR gate (mode: ordered-complex). The Trajectories column shows the number of trajectories corresponding to each reaction over the total number of trajectories simulated for the reactants.

Reactants	Products	Trajectories	k_1 ($M^{-1}s^{-1}$)	k_2 (s^{-1})
InA + OR	OR_InA_Waste + OR_InA_Sig	3163/103313	(1.67±0.04) $\times 10^6$	50.5±0.9
InB + OR	OR_InB_Waste + OR_InB_Sig	3136/61394	(2.88±0.07) $\times 10^6$	189±3
InA + OR	InA + OR	99856/103313	(4.71±0.02) $\times 10^7$	(5.28±0.25) $\times 10^3$
InB + OR	InB + OR	58258/61394	(5.02±0.02) $\times 10^7$	(5.04±0.42) $\times 10^4$

Table S13. Reaction rate estimates for the OR gate (mode: count-by-complex). The Trajectories column shows the number of trajectories corresponding to each reaction over the total number of trajectories simulated for the reactants.

Reactants	Products	Trajectories	k_1 ($M^{-1}s^{-1}$)	k_2 (s^{-1})
InA + OR	OR_InA_Waste + OR_InA_Sig	3159/106333	(1.63±0.04) $\times 10^6$	50.9±0.9
InB + OR	OR_InB_Waste + OR_InB_Sig	3121/62556	(2.80±0.07) $\times 10^6$	183±3
InA + OR	InA + OR	102874/106333	(4.71±0.01) $\times 10^7$	(5.02±0.23) $\times 10^3$
InB + OR	InB + OR	59435/62556	(5.02±0.02) $\times 10^7$	(4.76±0.37) $\times 10^4$

Table S14. Reaction rate estimates for the OR gate (mode: count-by-domain). The Trajectories column shows the number of trajectories corresponding to each reaction over the total number of trajectories simulated for the reactants.

Reactants	Products	Trajectories	k_1 ($M^{-1}s^{-1}$)	k_2 (s^{-1})
InB + AND	AND_InB	2583/6708	(1.26±0.03) $\times 10^7$	(1.13±0.03) $\times 10^7$
InA + AND_InB	AND_Sig + AND_Waste	3149/94505	(1.12±0.03) $\times 10^6$	60.3±1.1
InB + AND	InB + AND	4125/6708	(1.88±0.03) $\times 10^7$	(2.01±0.05) $\times 10^7$
InA + AND_InB	InA + AND_InB	91065/94505	(2.86±0.01) $\times 10^7$	(3.16±0.11) $\times 10^3$

Table S15. Reaction rate estimates for the AND gate (mode: ordered-complex). The Trajectories column shows the number of trajectories corresponding to each reaction over the total number of trajectories simulated for the reactants.

Reactants	Products	Trajectories	k_1 ($M^{-1}s^{-1}$)	k_2 (s^{-1})
InB + AND	AND_InB	3071/33859	(3.10±0.08) $\times 10^6$	(5.12±0.08) $\times 10^3$
InA + AND_InB	AND_Sig + AND_Waste	3149/97322	(1.09±0.03) $\times 10^6$	57.5±1.0
InB + AND	InB + AND	30788/33859	(2.85±0.02) $\times 10^7$	(2.85±0.14) $\times 10^5$
InA + AND_InB	InA + AND_InB	93865/97322	(2.87±0.01) $\times 10^7$	(3.20±0.11) $\times 10^3$

Table S16. Reaction rate estimates for the AND gate (mode: count-by-complex). The Trajectories column shows the number of trajectories corresponding to each reaction over the total number of trajectories simulated for the reactants.

Reactants	Products	Trajectories	k_1 ($M^{-1}s^{-1}$)	k_2 (s^{-1})
InB + AND	AND_InB	3066/36102	(2.89±0.07) $\times 10^6$	303±5
InA + AND_InB	AND_Sig + AND_Waste	3151/96431	(1.10±0.03) $\times 10^6$	57.2±1.1
InB + AND	InB + AND	33036/36102	(2.86±0.02) $\times 10^7$	(2.28±0.08) $\times 10^4$
InA + AND_InB	InA + AND_InB	93002/96431	(2.87±0.01) $\times 10^7$	(2.94±0.10) $\times 10^3$

Table S17. Reaction rate estimates for the AND gate (mode: count-by-domain). The Trajectories column shows the number of trajectories corresponding to each reaction over the total number of trajectories simulated for the reactants.
